# Quantifying Physical Activity and Sedentary Behavior in Adults with Intellectual Disability: A Scoping Review of Assessment Methodologies

**DOI:** 10.3390/healthcare12191912

**Published:** 2024-09-24

**Authors:** Cora J. Firkin, Iva Obrusnikova, Laura C. Koch

**Affiliations:** 1 Department of Health Behavior and Nutrition Sciences, University of Delaware, Newark, DE 19716, USA; obrusnik@udel.edu; 2 Temerty Faculty of Medicine, University of Toronto, Toronto, ON M5S 1A8, Canada; laur.koch@mail.utoronto.ca

**Keywords:** behavioral strategies, calibration, instruction, monitoring, support needs, wearable

## Abstract

**Background/Objectives**: Methodologies for assessing behavior form the foundation of health promotion and disease prevention. Physical activity (PA) and sedentary behavior (SB) assessment methodologies have predominantly been developed for adults without an intellectual disability (ID), raising credibility concerns for adults with ID. The purpose was to synthesize the current state of assessment methodologies for quantifying PA and SB volume in the free-living setting for adults with an ID. **Methods**: Following PRISMA guidelines, eleven databases were searched through December 2023, yielding 8174 records. Data were extracted in Covidence (v.2.0), obtaining quantified PA and SB volume and assessment methodology characteristics across data collection and analysis, including tool(s) and technique(s) used, preparatory actions taken, instructions provided, and behavioral strategies employed during data collection. **Results**: Of the 8174 articles screened, 91 met the inclusion criteria. Common metrics included minutes/hours per day/week and steps per day/week. Despite 80% of the studies using objective techniques, substantial variation existed across studies regarding wearable models, sampling frequency and epoch length settings, calibration protocols, wearable placements, and data processing techniques. Limited studies provided instructions that did not exclusively rely on spoken language. Behavioral strategies varied, including self-monitoring, providing assistance or supervision, administering questionnaires verbally, issuing reminders, and offering monetary incentives. **Conclusions**: This review underscores the need for greater consistency and accessibility in PA and SB assessment methodology for adults with ID. Tailored preparation, instruction, and behavioral strategies may enhance assessment viability and suitability for adults with ID, with or without caregiver or researcher involvement in the free-living setting.

## 1. Introduction

An intellectual disability (ID) is a developmental disability characterized by significant limitations in intellectual functioning and adaptive behavior, which encompasses the conceptual, social, and practical skills necessary for independent living, manifesting before 22 years of age [1]. Adults with ID experience declines in overall functioning [2] and adaptive behavior with aging [3] and face notable health disparities, such as a higher risk of cardiometabolic conditions, compared to adults without ID [4,5]. This “cascade of disparities”, often exacerbated by environmental and social factors, calls for urgent and comprehensive efforts to mitigate the onset and impact of chronic diseases and secondary health conditions in adults with ID [6,7], emphasizing healthier lifestyles through targeted behavioral modifications.

Physical activity (PA) and sedentary behavior (SB) are pivotal modifiable lifestyle factors influencing the risk of chronic diseases in adults without ID [8,9]. These behaviors can be categorized across educational, domestic, leisure, occupational, and transportation domains and have four dimensions—duration, frequency, intensity, and mode—collectively contributing to overall volume—the product of duration, frequency, and intensity [10,11,12,13]. PA is “any bodily movement produced by skeletal muscles that result in energy expenditure (EE)” [14] (p. 129) above a resting state, contributing to total daily energy expenditure. The intensity of movement is quantified using metabolic-equivalent-of-task values (METs), which represent the amount of oxygen burned at rest [15]. For adults, PA exceeds 1.5 METs and is categorized into three levels: light intensity (LPA, 1.6–2.9 METs), moderate intensity (MPA, 3.0–5.9 METs), and vigorous intensity (VPA, ≥6.0 METs) [13,15]. Consequently, SB, characterized by an EE of ≤1.5 METs, occurs during waking hours distinct from sleep [16]—a reversible neurobiological state characterized by behavioral quiescence, closed eyes, and perceptual disengagement from external environments [17]. In 2017, the Sedentary Behavior Research Network further detailed that SB occurs while sitting, reclining, or lying [16].

According to the 2020 WHO guidelines [13], each week, adults with or without disabilities, if capable, should accumulate 150–300 min of MPA, 75–150 min of VPA, or an equivalent combination of moderate-to-vigorous-intensity aerobic PA (MVPA), and at least two days of muscle-strengthening exercises targeting each major muscle group to achieve health benefits. Independent of PA levels [18], prolonged SB is associated with an increased risk of all-cause mortality and prevalence of cardiometabolic diseases [19]. Although the WHO has not yet established quantitative SB guidelines for adults with or without ID, they recommend all adults minimize SB and replace it with any type or intensity of PA, including LPA [13]. The only established quantitative SB guidelines are the Canadian 24-Hour Movement Guidelines for adults without ID [20], which recommend limiting sedentary time to eight hours and recreational screen time to three hours daily.

Nevertheless, as of 2020, the evidence of dose–response relationships between PA, SB, and health conditions for adults with ID was rated as “insufficient” [13]. Despite extensive synthesis across PA and SB research for this population, findings have been limited by notable methodological concerns, including insufficient datasets [21,22,23], inconsistent use of assessment tools [22], and the use of tools not validated or proven feasible, reliable, or sensitive specifically for assessing PA or SB in adults with ID [21,24]. Sallis’ 2000 Behavioral Epidemiological Framework [25] underscores the importance of aligning assessment methodology for behavioral research to establish clear links between behavior and health, marking essential initial phases of health promotion and disease prevention. A 2018 scoping review of PA research for adults with ID [24] revealed a critical need to enhance assessment methodology, noting that developing and refining methods comprised less than 10% of research.

In this review, PA and SB assessment methodology is operationally defined as a system of methods characterized by data collection and analysis protocols, including tools, techniques, preparatory actions and instructions, and behavioral strategies, all aimed at improving the viability and feasibility of assessing PA and SB. The selection of assessment tools and techniques directly influences the necessary preparatory actions, which are categorized into general actions (e.g., staff training), specific actions (e.g., tool development/refinement, calibration protocols, and device settings), instructions to participants or proxies, and behavioral strategies to increase targeted assessment task behaviors. Although indirect calorimetry and the doubly-labeled water technique are considered gold standards for EE assessment, no “perfect tool” or technique exists for PA [26] or SB [27,28] assessment.

PA and SB can be assessed using subjective techniques, which encompass indirect self- or proxy-reporting of behaviors via questionnaires, logs, diaries, and interview guides or checklists [10,26,29]. Conversely, objective techniques—favored by reviews [10,26,27,28,29]—primarily support using tools, such as accelerometers, inclinometers, heart rate monitors, pedometers, and multi-sensor devices, for more direct PA and SB quantification. These techniques require careful data processing considerations (e.g., software, non-wear-time definitions, cut-point parameters applied) for quality estimates. Notably, the first generation of ActiGraph, a prominent research-grade wearable brand [26,30], was instrumental in developing the original Freedson 1998 cut-point parameters [31], which classify activity intensities using uniaxial (y-axis) raw acceleration data converted to counts. Since 1998, additional cut-point parameters have been established, including Esliger 2011 classifications using raw acceleration data [32]. In 2022, ActiGraph released its algorithms for filtering and generating counts from raw acceleration data [33], allowing teams (e.g., [34]) to develop open-source packages for calculating counts from raw acceleration data from other wearables. However, proprietary algorithms to classify activity intensities are still common for consumer-grade (e.g., FitBit) and research-grade (e.g., PAL Technologies) devices. These evolving techniques underscore the dynamic nature of PA and SB assessment in research, setting the stage for continuous improvement and precision in methodologies from preparation to data processing.

### Purpose

Assessment methodologies for PA and SB have predominantly been developed for adults without ID, raising questions about the suitability [21], feasibility, validity, and reliability [24] of these tools and techniques to obtain sufficient data among adults with ID. An extensive, albeit non-systematic, literature search revealed that five systematic or scoping reviews have synthesized various facets of PA and SB assessment methodologies. The primary or secondary aims of these reviews focused on the tools and selected techniques for assessing PA [21,22,35] and SB [36] in individuals with ID, including children, and on selected strategies to boost compliance with wearing accelerometers for assessing PA [37]. However, these reviews, which completed their literature searches in [22,36,37] or before [21,35] 2015, did not cover the full range of methodological protocols necessary for assessing PA and SB in this population. Consequently, there is a critical need to synthesize recent advances and tailor assessment strategies to better meet the needs of adults with ID.

The objective of the present scoping review was to synthesize the current state of assessment methodologies for quantifying PA and SB in free-living settings for adults with ID. The review aims to identify (1) the data collection tools utilized and the techniques applied; (2) the preparatory actions and instructions provided; (3) the behavioral strategies employed by participants, caregivers, or researchers to enhance assessment data quality; and (4) the data processing and analysis techniques applied. A secondary aim is to outline the quantified PA and SB outcomes reported in the included studies and the reported data sufficiency metrics of the methodologies used to quantify PA and SB.

## 2. Materials and Methods

### 2.1. Protocol and Registration

The review adhered to the 2020 Preferred Reporting Items for Systematic Reviews and Meta-Analyses (PRISMA) guidelines [38] and incorporated the 2018 PRISMA Extension for Scoping Reviews. The protocol was registered in the International Database of Prospectively Registered Systematic Reviews on 7 October 2022 (reg. no. CRD42022351199).

### 2.2. Eligibility Criteria

The inclusion criteria for studies were: (1) participants with a mean age between 18.0 and 65.0 years old, or, if the mean age was not reported, a majority (>50%) of participants were within this age range; (2) over half of the participants explicitly reported to have an ID; (3) the use of a PA or SB assessment methodology that directly or indirectly quantified PA or SB volume in a free-living setting, defined as an uncontrolled, non-laboratory, non-simulated environment within the participants’ daily life activities; and (4) studies that were primary, peer-reviewed research published in full.

Studies were excluded if they: (1) only utilized qualitative assessments of PA or SB; (2) reported PA or SB outcomes as dichotomous or categorical variables (e.g., meeting guidelines or not); (3) reported fewer than three dimensions of PA or SB (e.g., frequency of activity modes or duration per bout); (4) involved examination of concurrent validity of multiple methodologies; (5) were conference proceedings, abstracts, editorials, dissertations, or theses; and (6) did not have the full text available in English.

### 2.3. Information Sources and Search Strategy

The search employed eleven electronic databases: CINAHL, Cochrane Library, ERIC via ProQuest and EBSCOhost, MEDLINE, PsycINFO, PubMed, Scopus, SPORT Discus with Full Text, Sports Medicine Education Index, and Web of Science. The initial search, up to 17 August 2022, was restricted to English-only, published peer-reviewed articles. The search strategy (Table A1) included terms related to the behaviors of interest (e.g., physical activity, sedentary behavior), target population (e.g., adult, intellectual disability), and quantitative assessment methodologies (e.g., quantify, measure), along with exclusionary terms. Searches were modified for each database using appropriate Boolean operators and database-specific filters (e.g., Human, English, Peer-Reviewed). Reference lists of relevant reviews (e.g., [21,22,35,36]) and included articles identified through the search were manually searched. An updated search covered articles published between 17 August 2022, and 31 December 2023, to include the entire 2023 calendar year.

### 2.4. Selection of Sources of Evidence

The first author (C.J.F.) imported search results into Covidence Systematic Review Software (v.2.0., Veritas Health Innovation. 2023. Melbourne, Australia). After duplicate removal using Covidence and manual checks, two authors (C.J.F. and I.O.) independently screened the titles and abstracts. Subsequently, they screened the full texts, moving forward only with articles where both authors agreed on eligibility. During the full-text screening, the authors excluded the articles using a drop-down box in Covidence, which included prepopulated reasons and allowed the authors to specify additional reasons for exclusion as necessary. Any disagreements in eligibility or reasons for exclusion were resolved through discussion. The inter-rater reliability of the screening was assessed by Cohen’s kappa coefficient [39]. The reference lists of relevant reviews and included articles were also screened using the same criteria.

### 2.5. Data Charting and Items

Data were extracted using the Covidence Data Extraction Tool, with items that were customized and pre-piloted by the authors. Articles were coded independently by the two authors (C.J.F. and L.C.K.). Any discrepancies were resolved through mutual consensus. Data items were guided by the recommendations outlined in the COSMIN Risk of Bias tool, which assesses the quality of studies on the reliability and measurement error of outcome measurement instruments [40]. Data items included article characteristics (e.g., publication year, data collection country, study design), sample characteristics (e.g., age, diagnoses), and assessment methodology characteristics (e.g., tools and techniques, staff training, tool development and refinement, tool set-up and calibration, instructions provided, behavioral strategies applied, data processing and analysis techniques). Also charted were the data sufficiency metrics where data were available (e.g., completeness, wear time) and the quantified PA and SB indicators (e.g., units and outcomes).

### 2.6. Collative, Summarizing, and Reporting Results

The number and percentage of articles that quantified PA, SB, or both in adults with ID were computed for the total sample of articles. Studies were categorized based on whether baseline indicators were PA or SB outcome(s) and whether data were collected by objective or subjective techniques. This categorization facilitated the summarization of their assessment methodology characteristics and data sufficiency metrics where data were available.

## 3. Results

### 3.1. Search Results

**Figure 1:**
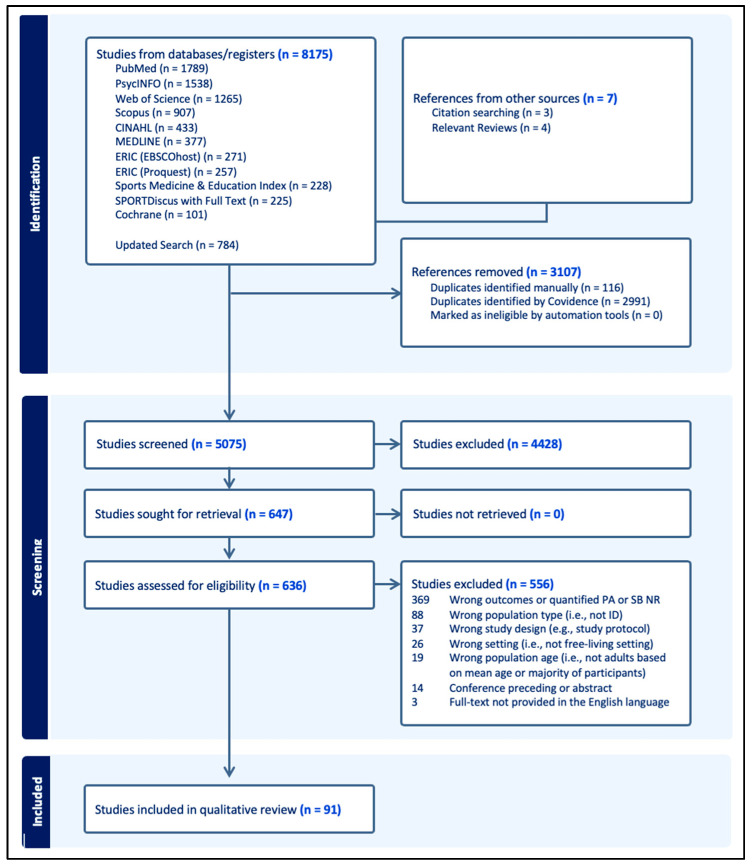
PRISMA Flowchart.

The search conducted on 17 August 2022, yielded 7391 results, from which 2882 duplicates were removed (Figure 1). From the remaining 4509 articles, 3923 were excluded based on title and abstract screening, and 506 following full-text screening, resulting in 80 articles. Additionally, seven articles identified through reference lists were added, bringing the total to 87 articles in the qualitative synthesis. An updated search on 20 February 2024, yielded an additional 784 results. After removing 225 duplicates, 505 articles were excluded during title and abstract screening, and 50 more were excluded after full-text review, resulting in an additional five articles. This brought the total number of articles for this review to 91. The overall inter-rater agreement (k) between the two authors (C.J.F. and I.O.) was 0.89 for the title and abstract screening, indicating an ‘almost perfect’ agreement, and 0.69 for full-text screening, indicating a ‘substantial’ agreement.

### 3.2. Source of Evidence Characteristics

The 91 included articles (detailed in Table A2) were published between 2000 and 2023, with 52 published in or after 2015. Study designs varied, encompassing cross-sectional (*n* = 53, 58.2%) [41,42,43,44,45,46,47,48,49,50,51,52,53,54,55,56,57,58,59,60,61,62,63,64,65,66,67,68,69,70,71,72,73,74,75,76,77,78,79,80,81,82,83,84,85,86,87,88,89,90,91,92,93]; non-randomized experimental [94,95,96,97,98,99,100] and randomized controlled trials [101,102,103,104,105,106,107] (each *n* = 7); case (*n* = 4) [108,109,110,111]; cohort (*n* = 3) [112,113,114]; qualitative (*n* = 2) [115,116]; and a study focused on method refinement [117]. Eight studies were secondary data analyses [118,119,120,121,122,123,124,125], and six were program evaluations [126,127,128,129,130,131]. Geographically, studies were predominantly conducted in the United States (*n* = 35, 38.5%), followed by the United Kingdom (*n* = 14), Spain (*n* = 7), Australia, Canada, and The Netherlands (each n = 5), France and Sweden (each *n* = 3), Hong Kong, Ireland, and Portugal (each *n* = 2), Denmark, Norway, South Africa, South Korea, Taiwan, and Poland (each *n* = 1). Two studies were multinational: Boonman et al. [45] included the Netherlands and United States, and Merzbach et al. [97] included Canada, Finland, Germany, Ireland, Myanmar, New Zealand, South Africa, Thailand, the United Kingdom, and the United States.

Data from 9458 adults with ID were synthesized from the reviewed studies, with sample sizes ranging from 2 to 1618 (M = 102.8, SD = 203.5). Fifteen studies exclusively involved adults with Down syndrome (DS), and three focused solely on adults with Prader–Willi syndrome. The age distribution of participants varied, with 11 studies reporting a mean age ≥18.0 to ≤25.9, 31 reporting a mean age ≥26.0 to ≤35.9, 37 reporting a mean age ≥36.0 to ≤45.9, seven reporting a mean age ≥46.0 to ≤55.0, and five reporting a mean age ≥56.0 to ≤65.0. The percentage of female participants ranged from 0% to 100%, with 43 studies reporting less than 50% female participants and 11 reporting exactly 50%.

### 3.3. PA or SB Volume Indicators

Most studies quantified volume indicators for exclusively PA (*n* = 53, 58.2%), followed by both PA and SB (*n* = 34), and exclusively SB (*n* = 4), as detailed in Table A2. The predominant metrics were minutes or hours per day or week for PA (*n* = 47) and SB (*n* = 33) levels and steps per day(s) or week (*n* = 33) for activity levels across sedentary to highly active lifestyles. Other metrics included intensity-based percentages per wear time, day, or week (PA: *n* = 11; SB: *n* = 12); MET-equivalents per day or week (PA: *n* = 10); counts per minute (CPM) per day or week (PA: *n* = 7); EE metrics, such as total daily or active EE per day or week (PA: *n* = 6); Physical Activity Level scores and steps per wear-hour or wake-hour (both *n* = 3). Nine studies reported indicators across all PA intensities and SB. No volume metric was reported for muscle-strengthening exercise. Six studies [43,53,59,73,97,112] reported the total frequency or count of participants participating in at least one bout of resistance training or weight training, and one [84] reported minutes per bout of calisthenics. Of the 38 studies reporting SB volume, four reported posture-related metrics, such as sitting or reclining time [88,97,111,124], and four reported recreational screen time, such as time spent watching television [59,70,71,121] and playing computer or video games [70,71].

### 3.4. PA and SB Assessment Methodology Characteristics

Across the 91 studies, 119 assessment methodologies were employed: 52 were solely objective, 38 were solely subjective, and 29 combined objective and subjective techniques. Assessment or recall timeframes varied, ranging from 5 to 28 days for solely objective techniques, 1 to 31 days for solely subjective techniques, and 2 to 14 days for combined techniques. Across all techniques, a seven consecutive-day timeframe was the most common (*n* = 78, 65.5%; Tables S1 and S2).

#### 3.4.1. Objective Tools

**Figure 2:**
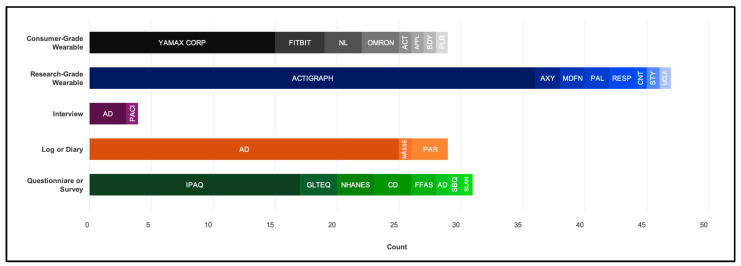
Assessment tools used to quantify physical activity and sedentary behavior in adults with intellectual disability, by brand or type, including ActivInsights Ltd. (ACT; Kimbolton, UK), ActiGraph LLC (Pensacola, FL, USA), Apple Inc. (APPL; Los Altos, CA, USA), Axivity Ltd. (AXY; Newcastle upon Tyne, UK), Body Media Inc. (BDY; Pittsburgh, PA, USA), CamNtech Ltd. and Inc. (CNT; Fenstanton, UK), Fitbit International Limited (Dubin, Ireland) and LLC (Mountain View, CA, USA), Muscle Dynamics Fitness Networks (MDFN; Torrance, CA, USA), New Lifestyles Inc. (NL; Lees Summit, MI, USA), Omron Healthcare Inc. (OMRON; Kyoto, Japan), PAL Technologies Ltd. (PAL; Glasgow, UK), Polar Electro Oy (PLR; Kempele, Finland), Respironics Inc. (RESP; Murrysville, PA, USA), Stayhealthy Inc. (STY; Monrovia, CA, USA), the UCLA Wireless Community (ULCA; Los Angeles, CA, USA), and Yamax Corp of Yamasa Tokei Keiki Co., Ltd. (Tokyo, Japan), or author derived (AD) or undetermined (CD).

Nearly 80% of the studies (*n* = 72) employed an objective technique. Only three studies (3.3%) used direct observations, with two of these [88,111] also utilizing wearables. Seven studies employed multiple wearable brands or models: three studies [84,85,86] used two compatible spring-lever pedometers of the same brand across the sample and one [74] used two compatible triaxial accelerometers, one [54] switched triaxial accelerometer brands after reported adverse reactions with the first, one [55] employed piezoelectric pedometers and triaxial accelerometers to obtain different outcomes, and one [130] employed triaxial accelerometers for participants who did not use a wheelchair and uniaxial accelerometers for participants who did. Wearables (detailed in Table S1) included triaxial accelerometers (*n* = 29), spring-lever pedometers (*n* = 17), piezoelectric (*n* = 6) pedometers, dual-axial accelerometers (*n* = 10), uniaxial accelerometers (*n* = 8), and heart rate monitors (*n* = 6). Some accelerometers could also support inclinometer functions (e.g., the ActiGraph models with the “inertial measurement unit function” enabled, Apple Watch via a gyroscope sensor, and activPAL). Three studies did not report the wearable model. The most common wearable brand was ActiGraph LLC (*n* = 36), formally, Manufacturing Technology, Inc. (Figure 2).

#### 3.4.2. Subjective Tools

Approximately 70% of the studies (*n* = 65) used a subjective technique (Figure 2). Questionnaires were the most common tool (*n* = 31), followed by logs or diaries (*n* = 30). Four studies employed multiple subjective techniques, such as linking logs with wearables and then employing recall questionnaires. The most common questionnaire was the International Physical Activity Questionnaire—Short-Form (*n* = 12), Proxy Report (*n* = 1), or an unspecified version (*n* = 4). For logs or diaries, recording frequencies varied, including intervals of every minute, 15, 30, or 60 min, or daily. If recordings based on the type and intensity of activity occurred every minute for seven days, this could potentially lead to as many as 20,160 entries. Across all subjective techniques (detailed in Table S2), the exact number of items recorded and by whom varied. Respondents included only participants (*n* = 22), both participants and proxies (*n* = 21), or only proxies (*n* = 17), with five studies not specifying the respondent type.

#### 3.4.3. General Preparatory Actions

Twenty-one studies (23.1%) did not explicitly report general or specific preparatory actions taken before PA or SB data collection (detailed in Table 1). General preparatory actions included conducting home or local venue visits to distribute assessment materials (*n* = 11); implementing blinding approaches to obscure assessment purposes, blinding either the assessor (*n* = 1) or the participant and/or caregiver (*n* = 10, e.g., sealing devices); acquiring expertise, gaining content knowledge, or performing supervised field training (*n* = 8); and organizing return logistics for tools, such as preparing pre-stamped, addressed envelopes (*n* = 5). In five studies [83,84,85,86,89], participants and, where applicable, caregivers were explicitly informed about the purpose of the tools, such as the pedometer count steps, to prevent tampering during the assessment.

#### 3.4.4. Specific Preparatory Actions for Objective Tools

Of the three studies using direct observations, one [41] reported that the assessment timeframe was chosen based on participant convenience, and another [111] prepared calendars of scheduled activities with caregiver assistance to facilitate seamless observer changeovers during the assessment timeframe.

Of the 71 studies involving wearables, the torso was the most common region for wearable placement, with 44 studies designating the hip or waist and one for the chest (Table S1). Seven studies designated wearable placement on the wrist, one on the upper arm, and three on the thigh. One study [62] gave participants the option to place the wearable either at the waist or either ankle. Furthermore, most studies (*n* = 45) did not specify the side of the body for wearable placement. Of those that did, 18 positioned the wearable on the “right”, seven on the “non-dominant”, and one on the “dominant” side, without detailing methods for determining dominance. Ten studies used specific anatomical landmarks or features for wearable positioning (e.g., iliac crest). Various apparatuses were used to secure wearables: belts, waistbands, wrist bands, elastic straps, pouches, clips, pant pockets, and adhesive pads. Eight studies reported that these apparatuses were custom-fitted, and 13 reported that researchers actively attached or placed the wearable on the participant’s body or belt.

Sampling frequency (Hz) was set at 30 (*n* = 4), 40 (*n* = 1), 60 (*n* = 3), or 100 (*n* = 3), with many studies (*n* = 47) not reporting this setting. Of those not reporting the frequency, wearables sampled at fixed frequencies of 10 (*n* = 2), 20 (*n* = 2), 30 (*n* = 8), 32 (*n* = 3), 100 (*n* = 5), or 1000 (*n* = 1). Other studies required setting sample frequencies within specific ranges: 30–100 (*n* = 14), 12.5–3200 (*n* = 2), or 0.7–5000 (*n* = 1). Epoch lengths were often set to 60 s intervals (*n* = 18) or adjusted (e.g., “re-integrated”, “summed”, “transformed”) to 60 s intervals from shorter intervals: 1 s (1), 5 s (*n* = 1), or 15 s (*n* = 4). However, 34 studies did not specify this detail. Where not specified, the wearable had fixed recording intervals, which included 4 s (*n* = 3), 5 s (*n* = 5), 10 s (*n* = 1), or 15 s (*n* = 3). Others (*n* = 6) required recording intervals to be set within a 1 to 60 s range. One study [53] set the wearable to its “Other Indoor” mode to capture data in 1 s intervals. Further setting details are available in Table S1.

Nine studies explicitly reported the wearable calibration practices: two [97,104] reported general calibration without providing the exact processes, and the others specified calibration procedures, such as performing a walking speed test, stride-length test [66,83,89], three-shake test [47], 30-step test [47], or graded step test [72] or 20 min outdoor walk [57]. Additionally, one study [87] reported preparing backup wearables for potential contingencies. For further support, two studies provided participants and caregivers with the researchers’ contact information for assistance. Most calibration and setup procedures were conducted by or in the presence of a researcher, except for one study [97] whose participants or caregivers set up the wearables at home, receiving virtual support as needed.

#### 3.4.5. Specific Preparatory Actions for Subjective Tools

Of the 65 studies employing subjective tools, four reported developing their own tool, and eight adapted an existing tool for their research. Of these, one study [97] pilot tested their newly developed tool, and four [70,71,112,117] assessed the validity, reliability, or suitability of their adapted tools. Two studies [68,121] prepared paper and online versions of their tools. For enhanced respondent comprehension, two studies [53,117] added written examples (e.g., “washing dishes as a household chore”), two [54,125] added pictures of activities on the tool, and one [88] provided proxy respondents with a completed example log. A total of 5 of 28 applicable studies reported the level of proximity between proxy respondents and participants, such as direct involvement in activities of daily living. Two studies verified participants’ understanding of assessment tasks by requesting examples to ensure clarity. Notably, one study [45] required participants to refrain from intense PA for 24 h before the assessment visit, during which the IPAQ was used to recall activities from the past seven days, a procedural choice that could potentially impact reported outcomes.

#### 3.4.6. Instructions Provided

Nearly 20% of studies (*n* = 17) did not report the instructional methods used to guide participants or caregivers on how or when to perform assessment tasks (Table 1). Instructional mode remained unspecified in 11 studies, with instructions noted simply as given or provided. For the studies that did provide details, instructions to participants most often involved direct instruction, such as verbal directives (*n* = 26, 28.6%, e.g., explicit commands) and verbal support (*n* = 34, 37.4%, e.g., encouragement). Indirect instruction included verbal explanations (*n* = 5, e.g., describing or explaining tasks) and written guidelines (*n* = 4), offering passive guidance without immediate feedback. Interactive instructional methods, which foster dynamic and reciprocal interaction, included visual demonstrations (*n* = 4) and video-enhanced instructions (*n* = 2), utilizing visual prompts through live or recorded actions. One study [88] developed video-based social stories. Another study utilized a visual activity schedule as a structured direct instruction tool to facilitate task performance. For caregivers, among the studies that provided instruction, direct instruction was the most common (*n* = 15; 16.5%), followed by verbal support (*n* = 8). Indirect instruction included verbal explanations (*n* = 7) and written guidelines (*n* = 7). Interactive instructional methods, such as visual demonstrations (*n* = 2), were less common but effectively utilized visual prompts to facilitate learning through observation.

#### 3.4.7. Familiarization with Assessment Tools

Familiarization with assessment tools was explicitly reported in only 10 studies (11.0%) for participants and two (2.2%) for caregivers (Table 1). This process typically involved supervised practice sessions (*n* = 7), during which participants learned how and when to use the assessment tools. During these, participants received individualized instruction [57,105] or extra practice sessions [88] as needed. Two studies allowed participants to use the wearables unsupervised at home to habituate to the device: one study [81] for a day before data collection and another [110] until activity levels stabilized. Familiarization for caregivers was formally addressed in one study [88], which implemented a standardized training program to ensure caregivers were adequately prepared to observe participants and included one or two practice observations with researcher assistance. The familiarization process was unspecified for two studies: one [72] noting participant familiarization with wearable calibration procedures, and the other [91] involving training caregivers on how to re-secure the wearable as needed.

#### 3.4.8. Behavioral Strategies during Data Collection

Twenty-three studies (25.3%) did not specify behavioral strategies undertaken by the participants, caregivers, or researchers during data collection (Table 1). Among the reported strategies undertaken by participants, self-monitoring was the most prevalent (*n* = 20; 22.0%). Of these studies, five employed logs or diaries to monitor activity information (e.g., mode) [53,57,59,73] or wear times or unusual occurrences [57,59,79], with these details not used for PA or SB volume outcomes. Other participant strategies included environmental modifications and behavioral supports (*n* = 7), such as choosing preferred wearable placements [54,55,62,78,109], carrying the log on one’s body throughout the day [88], and storing the wearable in a designated cup when not in use [109].

In 23 studies, caregivers were instructed to assist, with 17 specifying assistance as needed, and 2 [52,110] reporting that caregivers supervised participants during data collection tasks. Caregivers also monitored participants in 25 studies, with 6 employing logs or diaries to obtain activity information [53,57,59,73] and wear times or unusual occurrences [57,59,79,90] not directly used for calculating PA or SB volume outcomes. Other caregiver strategies involved establishing conditions to facilitate proper data collection (*n* = 11), such as removing or resetting wearables (e.g., before water-based activities or the wake period) [72,83,84,86,87], providing reminders [52,87,109,110], and managing medical records [70]. For proxy respondents, strategies also included environmental modifications and behavioral supports (*n* = 3), such as offering a choice between written or online questionnaire format [68,121] and aligning log recording with mealtimes to promote consistency [88]. Two studies reported caregivers were directed to provide negative feedback to discourage tampering with the wearables to artificially inflate step counts. One study required caregivers to verify log entries before data analysis to ensure accuracy.

Researcher strategies primarily involved establishing conditions to facilitate proper data collection (*n* = 29, 31.9%). These strategies included providing reminders and cues (*n* = 11): verbally (e.g., during in-person interactions or phone calls) [93,109]; via visual displays (e.g., posters or picture cards) during tasks [69,73]; and via text-based phone messaging [59,81,87,88,118] or online posts [68,97]. Additionally, researchers administered questionnaires verbally (*n* = 10) [56,60,61,70,71,77,84,106,122,125]; managed the extraction or recording of wearable data (*n* = 8) [53,62,88,90,100,109,110,111]; and preemptively switched wearables before and after work to ensure proper data classification (*n* = 1) [47]. Thirteen studies reported that researchers assisted or supervised participants, including clarifying instructions [61,82,84,93], ensuring wearables were properly put on [52,59,104] or taken off and charged [51,53,59,109], and verifying log or diary entries were accurately completed [53,57,59,65,66]. Three of these studies [57,65,66] utilized wearable memory to facilitate log completion. Researchers also monitored participants in five studies, with two [57,59] recording participants’ activity information and wear times or unusual occurrences in logs or diaries with these details not directly used for calculating PA or SB volume outcomes. Director observers timed log recording every minute in one study [111] and every 15 min in another [41] to accommodate varying study designs. In seven studies, the assessment timeframe was modified as needed [109,110] or extended to a set number of days [52,53,74,75,79] to ensure sufficient data collection. Six studies reported researchers collected the tools after the assessment timeframe at locations convenient for participants. Furthermore, nine studies reported providing monetary incentives ranging from 5.00 to 100.00 USD. However, these studies did not specify whether payments were contingent upon participants’ compliance with data collection protocols.

#### 3.4.9. Objective Data Processing Techniques

In 71 studies employing wearables (Table S1), data processing typically involved the wearable brand-specific software, including ActiLife version.5.0-6.13.4 (*n* = 11), and ACTi4, FlowSync version.6.7.0, GENEActiv version.NR, Omron Health Management version.NR, and Sensewear Pro version.6.2 (each *n* = 1). Additionally, nine studies used customized packages, programs, or servers for data processing. Most studies (*n* = 48, 67.6%) did not specify the data processing software used or define non-wear-time parameters (*n* = 51, 65.4%). Eight studies referenced specific non-wear-time algorithms, with seven using the Troiano 2007 [132] algorithm, one using Choi 2011 [133], and another Cain 2018 [134]. Fifteen studies defined non-wear time, with five allowing brief interruptions and four without such allowances. Three studies supplemented these with log or diary records. The minimum wear-time compliance criteria to ensure sufficient data varied from one to seven days per week, with daily wear-time requirements ranging from no specified minimum up to the full 24 h. Common weekly wear-time compliance criteria were at least three (*n* = 16) or four (*n* = 20) days with minimum daily wear times of eight (*n* = 8) or ten (*n* = 19) hours. Eighteen studies required at least one weekend day, with eight requiring both Saturday and Sunday. Although five studies required wear on all assessment days, they did not specify a minimum number of

To classify PA and SB, 36 studies explicitly referred to previously identified cut-point parameters (Table S1), including the Freedson 1998 Adult (*n* = 11) or Modified Version (*n* = 5); Troiano 2008 Adult Version (*n* = 13); Atkins 2012 Adult Version (*n* = 3); Agiovlasitis 2022 Vector Magnitude Version for Adults with DS, Esliger 2011 Raw Accelerometer Data Version, Kim 2015 Children Version, and Peiris 2016 Version for Young Adults with DS (each *n* = 1). Therefore, applied count-based uniaxial (‘y’) cut-point parameters (in CPM) varied for SB, including 0–15 (*n* = 1), 0–100 (*n* = 36), and 0–500 (*n* = 4); LPA, including 100–1921 (*n* = 11), 100–2019 (*n* = 14), and 500–1921 (*n* = 5); MPA, including 1389–2448 (*n* = 1), 1952–5724 (*n* = 16), and 2020–5998 (*n* = 14); and VPA, including 2449–∞ (*n* = 1), 5725–∞ (*n* = 16), 5999–∞ (*n* = 13), and 5999–20,000 (*n* = 1). The applied vector magnitude parameter for SB was 0–236. One applied defined MET-min parameters for SB (≤1.49), LPA (1.5–2.99), MPA (3–5.99), and VPA (≥6). Three studies [59,94,118] explicitly excluded PA bouts less than 10 min from analysis. The Tudor-Locke 2004 standard was mainly applied in 13 studies to assess overall activity levels based on daily step counts. One study [114] categorized MVPA based on a minute-level cadence of ≥109 steps. Notably, 13 studies did not report cut-point parameters and 16 did not report a step-count index, highlighting a gap in the reporting that could affect the interpretability of findings across studies.

#### 3.4.10. Subjective Data Processing Techniques

Subjective data processing techniques, such as transcription and coding, were infrequently detailed across the 65 applicable studies. Two studies [53,57] used Microsoft Excel to compile and organize subjective data. Intensity categories were coded in two studies [112,114] that employed the 2003 Scottish Health Survey definitions for LPA (“gentle walking or light gardening”), MPA (select activities “if the person does not become sweaty or out of breath”), and VPA (select activities “sufficient to make the person breathe or sweat heavily”). One study [93] used “alternative language” to classify activity intensities during interviews, labeling them as LPA (“easy”), MPA (“somewhat hard”), and VPA (“hard”) minutes. Five studies [70,71,82,117,125] explicitly reported the methods used to transform or calculate variables (e.g., summing each duration spent in different activity modes). MET scales were referred for coding activity intensities, including the IPAQ’s 3-point scale (*n* = 5; [45,60,69,77,103]), Bouchard’s 1983 PAR’s 9-point scale [41,88,111] and Godin’s 1985 GLTEQ’s 3-point scale [48,49,50] (both *n* = 3), and the 1993 Ainsworth Compendium for Physical Activities (*n* = 2; [56,84]). Two studies [60,106] referred to the IPAQ-SF scoring protocol to calculate weekly minutes of PA and SB. One study [57] specifically coded log data to calculate the percentage contribution of each respondent type: participant, caregiver, or researcher.

### 3.5. Data Sufficiency Metrics

The proportion of participants with sufficient PA or SB data for analysis ranged from 24.5 to 100.0% for objective techniques, 0 to 100% for subjective, and 24.5 to 100.0% for combined techniques (Table A2). The PA or SB data sufficiency also varied by wearable type, ranging from 44.6 to 100% for research-grade and 24.5 to 100% for consumer-grade devices. Specific wearable types showed varied sufficiency rates: 58.3–100% for triaxial accelerometers, 24.5–100.0% for spring-lever pedometers, 33.3–93.3% for piezoelectric pedometers, 44.6–64.7% for uniaxial accelerometers, 64.3–100% for dual-axial accelerometers, and 66.1–100% for heart rate monitors or multi-sensors. Wearable placement also influenced data sufficiency, with chest placements showing a 93.3% sufficiency rate, wrist placements from 66.7 to 100%, thigh placements 66.1 to 86.4%, and hip or waist placements ranging from 24.5 to 100%. Only 22 studies (24.2%) reported descriptive wear time statistics, with daily wear times ranging from 9.6 to 21.4 h. Half of these studies (52%, *n* = 13) reported daily wear times of 12.7 h or less. By wearable placement, the average daily wear time was 20 h for the chest and 16 h for the upper arm, varied from 11.3 to 21.4 h for the wrist, and 9.6 to 14.5 h for the hip or waist. No wear-time data were reported for thigh placements in three studies. Similarly, for subjective tools, data sufficiency rates varied from 11.8 to 100% for logs or diaries, 0.0 to 100% for questionnaires, and consistently 100% for interviews. By respondent type, sufficiency ranged from 16.7 to 100% for self-reported data, 13.3 to 100% for proxy reports, and 11.8 to 100% for mixed respondents. However, 31 studies (34.1%) did not provide sufficient information to determine data sufficiency.

## 4. Discussion

The present scoping review analyzed 91 articles that met the inclusion criteria to synthesize assessment methodologies for quantifying PA and SB in adults with ID. The findings highlight a diverse application of tools, techniques, preparatory actions, instructions, and behavioral strategies, with approximately 80% of studies utilizing at least one objective technique and 70% utilizing at least one subjective technique. Notably, methodologies combining both objective and subjective techniques, such as integrating logs with accelerometers, yielded richer data and a more comprehensive understanding of participants’ behaviors. The review also uncovered significant variability in data sufficiency metrics, highlighting the challenges in achieving consistent and reliable PA and SB assessments for adults with ID. This variability underscores the urgent need for standardized assessment protocols and clearer reporting practices to enhance the accessibility and utility of these techniques, both when used exclusively and in combination with other techniques. 

A critical finding of this review is the considerable variation in PA and SB outcome units across studies, highlighting a crucial gap in standardized terminology and reporting practices. Establishing consistent metrics would facilitate comprehensive comparative analyses and, thereby, enhance a more robust understanding of the impacts of PA and SB, especially across diverse—and evolving—assessment tools [26]. Such standardization is essential for public health surveillance [5,27,135] and the development of PA and SB guidelines supported by a cumulative, homogenous body of evidence on the dose–response relationships between PA, SB, and health outcomes for adults with ID [13]. Current guidelines for adults are expressed in weekly minutes of MPA and VPA [13,136] or combined MVPA [20] and daily hours of SB [20]. Therefore, it would be beneficial for future research to report PA and SB outcomes in these units to align findings with actionable guidelines. In accordance with Pate’s 2008 recommendations [137], these outcomes should encompass all activity intensities, including SB, LPA, MPA, VPA, and combined MVPA. However, only 37.3% of studies in this review quantified a PA and SB indicator, with only 9.9% of studies reporting across all intensities of PA and SB. In addition, current guidelines include thresholds for muscle-strengthening exercise [13,20,136] and recreational screen use [20]. Nonetheless, this review reveals a scarcity of reported volume of muscle-strengthening and recreational screen time activities. Consistent assessment of these activities is crucial to guide and support recommendations for adults with ID, indicating an urgent need for more comprehensive research in this area.

Likewise, the findings emphasize the need for careful articulation of operational definitions and PA and SB outcome units to ensure that they align with quantified metrics [11,27,36,137,138], particularly those quantifying behaviors exclusively in specific postures or activity modes. Distinguishing between different postures and activity modes remains challenging across the PA and SB field, whether using accelerometers, heart rate monitors, or inclinometers. For example, thigh-worn inclinometers, such as activPAL, can assess postural changes but cannot fully distinguish between sitting and lying or between passive and active non-ambulatory postures (e.g., squatting vs. sitting) [28]. Similarly, passive and active sitting is challenging to distinguish with accelerometry [10]. These challenges are particularly relevant because postures and activity modes may cause diverse metabolic responses in adults with ID. For instance, according to Lante’s 2010 findings [139], adults with ID, including those with and without DS, expend significantly more energy than adults without ID in several activities, such as standing and sitting quietly, watching television, or while performing an assembly task. Notably, EEs for passive sitting behaviors for adults with ID are all above the SB threshold of 1.5 METs [16]. Furthermore, devices like the activPAL attribute 1.25 METs to both sitting and lying positions and 1.40 METs to standing by default [28]. Caution is warranted when employing wearables that use proprietary algorithms to classify PA and SB intensities based on postures or activity modes, as these contractions may not accurately reflect the metabolic costs in adults with ID.

Moreover, there is a crucial need to validate data processing techniques for adults with ID to ensure precise characterization of PA and SB, which is particularly important for correctly differentiating LPA, SB, and sleep [37]. This review highlights a notable scarcity in the application of population-specific cut-points, with only two studies [42,81] using parameters specifically developed for adults with DS and one [42] using a tri-axial (vector magnitude) cut-point parameter that more effectively estimates EE for adults with DS than uniaxial parameters [140,141]. These parameters were developed for hip or waist-worn ActiGraph wearables similar to prominent parameters for adults without ID (e.g., Freedson 2008) [26,31]. Building on Leung’s 2017 review [37], the findings revealed a greater proportion of participants achieving sufficient PA or SB data when using wrist-worn wearables. For adults without ID, recent trends toward smaller, wrist-worn wearables have led to significant advancements in processing raw acceleration data [142]; however, these techniques for converting raw accelerometry into counts often remain inaccessible to researchers [143]. In this review, only one study [54] utilized Esliger’s raw accelerometry parameters. To enhance the clarity, transparency, and utility of PA and SB data, detailed accounts of all data processing techniques should be included in research publications or supplemental materials [37,135,138]. Furthermore, making raw and processed data accessible in public repositories would facilitate the application of evolving processing techniques and support more homogenous data accumulations and comparisons over time [37,135].

These recommendations parallel those for subjective data processing, which were also infrequently detailed in this review. Nonetheless, all MET scales used in the applicable studies in this review were originally developed for adults without ID, thus potentially leading to inaccurate estimations due to expenditure differences [139]. Additionally, the studies employed questionnaires with recall periods ranging from the past day to the past month, with 45.2% of these recalls completed exclusively by participants with ID. Yet, only five studies [54,70,71,117,125] made changes to instructions or questionnaire items. Although these changes were intended to facilitate comprehension, it remains unclear whether these changes were comprehensive adaptations to match the unique cognitive profiles of participants with ID or merely adjustments. This review identified that questionnaire data sufficiency varied from 0.0 to 100%, with two studies [103,127] omitting to report their questionnaire data due to considerable data quality concerns. This warrants the urgent need to carefully adapt questionnaires to be relevant, feasible, and valid for adults with ID, and develop strategies to enhance the accuracy of mixed respondent participation involving both participants and their caregivers.

Given the limitations of single tools in accurately assessing PA and SB, incorporating multiple complementary tools is crucial for a comprehensive, multifaceted representation of PA [11,12] and SB [28], especially in studies involving adults with ID [21]. The integration of multi-sensor devices or various objective techniques offers a more nuanced approach but introduces complexities, such as the need for compound data harmonization models that rely on proprietary or complex algorithms (e.g., machine learning) to classify PA and SB [28,138,143]. Linking logs or diaries with timestamps from wearable devices can help clarify postures and activity modes and exclude non-wear and sleep times, potentially inflating SB metrics [28]. This review identified varied data sufficiency for both wearables (24.5–100.0%) and logs or diaries (11.8–100%). Data sufficiency varied widely for wearables (24.5–100.0%) and logs or diaries (11.8–100%). Moreover, daily wear times averaged approximately 12.7 h or less, suggesting that wear times might cover only approximately 70–80% of the estimated 16–18 h waking period for adults [17]. However, only 24.2% of the applicable studies reported detailed wear-time data, and very few provided data on wake periods, necessitating standardized reporting of wear times and wake periods alongside daily PA and SB outcome metrics. This variability warrants further investigation into making these tools more accessible and usable by participants and their proxies in unsupervised, free-living settings.

To achieve these improvements, this review emphasizes the critical need for robust methodological reporting in studies involving adults with ID. This includes detailed descriptions of preparatory actions, instructions, and familiarization strategies before data collection, alongside behavioral strategies during data collection, all aimed at enhancing the suitability and accuracy of the research. General preparatory actions, such as training research staff to ensure proficiency in tool usage and understanding participant support needs, implementing blinding protocols to minimize bias, and organizing visits to distribute assessment materials to participants, were infrequently reported. Similarly, specific preparatory actions for wearables and subjective tools lacked detail, which may impact data quality [30]. Across studies were considerable variations in wearable selection, placement, epoch length, and sampling frequency; only nine studies detailed calibration techniques, which may impact wearable precision [144]. This review recommends comprehensive reporting of preparatory actions to enhance replicability [30].

Moreover, the review strongly advocates for research protocols that are accessible and carefully designed to meet the support needs of adults with ID. It is recommended to expand instructional strategies beyond verbal commands to enhance participant and caregiver comprehension of the assessment tasks. For instance, Matthews et al. advocate for written instructional sheets with visual aids for proper wearable positioning and device care [135]. Similar to Leung’s 2017 review [37], studies in this review that incorporate visual aids, interactive demonstrations, and customized familiarization protocols to address various learning styles have achieved higher data sufficiency (e.g., 85.0–100% compared to 24.5–100.0% for exclusively objective techniques). However, a significant gap persists in the systematic implementation of these instructional strategies before data collection. This review also recommends thoroughly detailing the behavioral strategies employed to enhance the clarity, consistency, uniformity, replicability, and transparency of PA and SB research involving adults with ID. Such detailed reporting is critical for advancing the practical and effective application of assessment methodologies tailored to this population, ensuring high data sufficiency and quality [37]. The review further encourages journals to allow more extensive methods sections and the inclusion of supplemental materials to elaborate on these methodologies. Additionally, it suggests exploring further customizable approaches that effectively meet the support needs of adults with ID without compromising methodological integrity.

### 4.1. Limitations 

Similar to Dairo’s 2016 systematic review [22], this scoping review did not attempt to locate unpublished studies. Thus, the potential for publication bias remains unassessed. Nevertheless, the observed negative skew in data sufficiency metrics suggests a potential publication bias, where studies with sufficient data may be preferentially published. In addition, not all databases were searched [37]. These biases could present an incomplete picture of the scope of assessment methodologies used for quantifying PA and SB in adults with ID. Additionally, this review did not perform a quality assessment of the included studies, which prevented the identification of systematic weaknesses in the existing research. Thirty-one studies did not provide sufficient information to determine data sufficiency, which is a considerable concern for not only assessments but also PA interventions [30]. Consequently, the findings should be interpreted with caution.

### 4.2. Research Gaps and Recommendations

This review has identified significant gaps in the validity and reliability of assessment tools used with adults with ID, echoing findings from previous reviews [22,35,36]. Addressing these gaps necessitates developing robust validation processes, starting with establishing clear and consistent definitions of PA and SB [11]. For example, although the postural-based SB definition proposed by the 2017 Sedentary Behavior Research Network offers a valuable framework, it lacks specific face and content validity for adults with ID [16]. To effectively capture PA and SB, methodologies must be comprehensive enough to fulfill the research objectives and adaptable to the target population’s capabilities [11]. This involves customizing preparatory actions, instructions, and behavioral strategies to ensure that participants and, where necessary, their caregivers fully understand and engage with the data collection tasks, minimizing measurement errors and maximizing data reliability. Before advancing to more complex validation stages, such as establishing concurrent or predictive validity, these foundational efforts must address feasibility and establish face and content validity [11]. Moreover, this review advocates for a paradigm shift in the methodological frameworks used in PA and SB research involving adults with ID. It calls for methodologies that are inclusive and adaptable, where feasible, to meet the complex needs of this population. Such a shift is crucial for enhancing the effectiveness of PA and SB assessments and, thus, enhancing the quality of data used to inform strategies aimed at improving the health and well-being of adults with ID [25].

## 5. Conclusions

The quality of behavior assessment is fundamental in research [25], where accurate data on PA and SB are critical for effective disease prevention and health promotion involving individuals with ID. Previous reviews [24,37] have stressed the necessity of uniform assessment protocols for adults with ID to secure high-quality PA and SB data that can inform health strategies at both individual and population levels. However, the rapid technological advancements and innovations present significant challenges to maintaining such uniformity. This scoping review calls for comprehensive and standardized reporting practices for PA and SB indicators and units, along with protocols that are systematically implemented both before and during data collection. Implementing these practices will ensure that PA and SB assessment methodologies are consistent and accessible for adults with ID, thereby improving the quality and applicability of research outcomes.

## Figures and Tables

**Table 1:** The preparatory actions, instructions, and strategies reported in physical activity and sedentary behavior assessment methodologies for adults with intellectual disability (*n* = 91).

		**ID**	**%**
Preparatory Actions Before Data Collection			
General	Conducted a Local Venue Visit	2, 16, 18, 24, 47, 67, 69, 81–83, 87	12.1
	Blinded PPT, CG, or Researcher	10, 19, 20, 25, 27, 52, 57, 66, 81, 82	11.0
	Trained Research Staff	3, 17, 19, 54, 55, 74, 81, 90	8.8
	Organized Logistics for Tool Return	18, 45, 58, 67, 73	5.5
Direct Observation	Planned for Observer Changeovers	1, 81	2.2
	Chose Convenient Timeframe for PPT	1	1.1
Wearables	Designated Wearable Placement(s) ^†^ and apparatus(-es) to secure it in place	2, 3, 4 ^†^, 5 ^†^, 7 ^†^, 10, 14–16 ^†^, 18 ^†^, 19, 21 ^†^, 24–27 ^†^, 30, 31 ^†^, 33 ^†^, 34 ^†^, 36–38 ^†^, 40 ^†^, 42 ^†^, 43 ^†^, 48, 49 ^†^, 50, 51 ^†^, 53 ^†^, 56–58 ^†^, 60 ^†^, 62, 63, 65–67 ^†^, 69 ^†^, 70 ^†^, 72, 73 ^†^, 75–82 ^†^, 85 ^†^, 87–89 ^†^	61.5
	Reported Epoch Length Setting	2, 14–17, 19, 26, 31, 33, 34, 40, 50, 51, 56, 58, 60–61, 63, 65, 67, 69–70, 73, 89	26.4
	Placed Wearable on PPT Belt or Body	27, 36, 38, 49, 56, 65, 67, 71, 75, 77–79	14.3
	Calibrated Wearable	10, 25, 36–38, 53, 56, 76, 83	9.9
	Custom-Fit Wearable Bands	2, 14, 16, 27, 31, 62, 63, 67	8.8
	Reported Sampling Frequency Setting	2, 14, 26, 31, 58, 61, 65, 89	8.8
	Chose Long Timeframe	16, 37, 60	3.3
	Provided Contact Information	18, 80	2.2
Subjective Tools	Adapted or Altered Existing Tool	3, 16, 18, 27, 45, 54, 55, 78, 81, 82, 86, 87	13.2
	Developed Tool or Instrument	23–25, 39, 53, 91	5.5
	Identified Proximity of the Proxy	29, 46, 54, 55, 78	5.5
	Prepared Examples of or on Tools	16, 18, 82, 86, 87	5.5
	Prepared Paper and Online Version	39, 45	2.2
	Verified PPT Understood Tasks	16, 65	2.2
Provided Instructions Before Data Collection To:			
PPT	Verbal Support	1, 4, 5, 14, 20, 23, 25, 38, 40, 46, 48–53, 55, 60, 65, 67, 69–71, 75, 77–80, 83–85, 87, 89, 90	37.4
	Verbal Direct Instruction	2, 3, 5–7, 9, 16, 18, 25, 26, 30, 43, 49, 50, 53, 57, 58, 63, 66, 67, 76–79, 83, 85	28.6
	Verbal Explanation	20, 24, 65, 80, 82	5.5
	Written Guidelines	2, 63, 67, 80	4.4
	Visual Demonstration(s)	18, 42, 82, 83	4.4
	Video-Enhanced Instruction	53, 82	2.2
	Visual Activity Schedules	25	1.1
CG	Verbal Direct Instruction	5, 15, 16 *, 36, 37, 48, 63, 67, 76–79, 82, 83, 88	16.5
	Verbal Support	1, 12, 40, 67, 75–76, 78–79	8.8
	Written Guidelines	2, 45, 63, 67, 72, 80, 82	7.7
	Verbal Explanation	20, 46, 56, 65, 80, 82	6.6
	Visual Demonstration(s)	42, 83	2.2
Familiarization With Assessment Tools Before Data Collection For:			
PPT	Practice in Supervised Setting	16, 25, 27, 48, 81–83	7.7
	Habituate in Unsupervised Setting	57, 73	2.2
CG	Practice in Supervised Setting	82	1.1
Behavioral Strategies Undertaken During Data Collection By:			
PPT	Self-Monitored in Log or Diary	9, 16 ^¶^, 25 ^¶^, 26, 27 ^¶^, 44, 46, 47, 51, 52, 58 ^¶^, 67 ^¶^, 77–84	22.0
	Environmental Modification/Behavioral Support	18–19, 30, 43, 53, 66, 82	7.7
CG	Provided Assistance or Supervision	5 *, 9, 15, 18 *, 19, 22 *, 24 *, 27 *, 29 *, 33 *, 38 *, 51 *, 52 *, 54–57, 65 *, 68 *, 75 *, 78 *, 80, 86 *, 87 *, 91 *	27.5
	Monitored in Log or Diary	1, 9 *, 16 *^¶^, 17, 25 *^¶^, 26, 27 *^¶^, 36, 37, 46, 47, 51, 52, 58 ^¶^, 67 ^¶^, 76, 78–83, 85 ^¶^, 88, 89	27.5
	Establishing Conditions to Facilitate Data Collection	15, 39, 43, 45, 54, 56, 57 *, 76, 78–80	12.1
	Environmental Modification/Behavioral Support	39, 45, 82	3.3
	Provided Negative Feedback	76, 78	2.2
	Verified Log Entry Accuracy	27	1.1
Researcher	Establishing Conditions to Facilitate Data Collection	7, 10, 16, 22–24, 27 *, 28–30, 43, 45, 46, 52–55, 57, 58, 65, 73, 78, 80–82, 85, 87, 90, 91	31.9
	Provided Assistance or Supervision	14–16 *, 25 *, 27 *, 29, 36–38, 43, 74, 78, 91	14.3
	Provided Monetary Incentives	3, 16, 19, 22, 25–26, 29, 48, 89	9.9
	Modified Assessment Timeframe	15–16, 43 *, 57 *, 60, 62, 67	7.7
	Collected Tool at Convenient Locations for PPT/CG	40, 48, 49, 63, 85, 87	6.6
	Monitored in Log or Diary	1, 10, 25 *^¶^, 27 *^¶^, 81	5.5
	Environmental Modification/Behavioral Support	1, 81	2.2

Notes. CD: cannot determine; CG: caregiver; ID: study identification number (see Table A2); PPT: participant. * As needed. ^†^ Only designated wearable placement and not apparatus for securing into place. ^¶^ Not used for PA or SB volume.

## Data Availability

The data supporting this study’s findings are available in supplemental materials and from the corresponding author upon reasonable request for verification or collaboration purposes.

## Supplementary Materials

The following supporting information can be downloaded at: https://www.mdpi.com/article/10.3390/healthcare12191912/s1, Table S1: Wearable specifications to quantify physical activity and sedentary behavior in adults with intellectual disability, by the manufacturer; Table S2: Subjective techniques used to quantify physical activity and sedentary behavior in the included studies. References [145,146,147,148,149,150,151,152,153,154,155,156,157,158,159,160] are cited in the Supplementary Materials.

## Appendix A

**Table A1:** Full search strategy.

Sample	Physical Activity or Sedentary Behavior	Methodology/Assessment	Exclusions
(adult*) AND (“intellectual* disab*” OR “intellectual* disorder*” OR “intellectual* impair*” OR “mental* retard*” OR “cognitive* deficien*” OR “cognitive* disab*” OR “cognitive* handicap*” OR “cognitive* subnormal*” OR “cognitive* impair*” OR “development* disab*” OR “development* disorder*” OR “development* impair*” OR “Down* syndrome” OR “prader willi” OR prader-willi OR trisomy OR “Special Olympic*” OR “learning disab*” OR “learning impair*”)	(“phys* activ*” OR “energy expend*” OR “daily life activ*” OR “free-living activ*” OR aerobic* OR muscle OR strength OR “leisure activ*” OR exercis* OR “motor activ*” OR fitness OR “life-style activ*” OR “life style activ*” OR “phys* behav*” OR “sedentary behavior” OR “sedentary” OR sitting OR lying OR reclining)	(method* OR assess* OR measure* OR protocol OR test* OR subjective OR objective OR surveillance OR screen* OR classif* OR regulat* OR self-regulat* OR tool OR device OR wearable OR track* OR monitor* OR calorimet* OR acceleromet* OR pedomet* OR actiwatch OR smartwatch OR “motion logger” OR “smart tech*” OR “smart watch” OR “smart phone” OR smartphone OR “doubly label*” OR survey OR questionnaire OR log OR diary OR report OR interview OR recall OR scale OR index* OR indices OR application OR observation* OR strateg* OR instruction OR prompt* OR familiarization OR psychometr* OR feasibl* OR valid* OR reliab* OR sensitivity OR specificity OR “measurement accuracy” OR “measurement error” OR “measurement precision” OR “measurement effectiveness”)	(child* OR infant* OR toddler* OR pediatric* OR stroke OR Parkinson* OR Alzheimer* OR dementia* OR “brain injury” OR “hearing impair*” OR deaf OR blind OR “mobility impair*” OR “physical impair*” OR dystroph* OR “multiple sclerosis” OR “fatigue syndrom*” OR HIV* OR schizophren* OR illness* OR fracture* OR injur* OR pregnan* OR frail* OR animal* OR mice OR vaccin*)
Database	Additional Limitations or Filters		
CINAHL	Scholarly Peer Reviewed, English Language, Journal Article, English		
ERIC (Proquest)	Peer Reviewed, Exclude duplicate documents, English, Journal Articles		
MEDLINE	Peer Reviewed, Exclude duplicate documents, English, Journal Articles		
PsychINFO	Peer Reviewed, Exclude duplicate documents, English, Journal Articles		
SportsMedicine & Education Index	Peer Reviewed, Exclude duplicate documents, English, Article		
SPORTDiscus with Full Text	Peer Reviewed, English abstract available, English Language, Article		
PubMed	English, Journal Article, Humans		
Scopus	English, Article, Journals, Final Publication Stage, Human		
Cochrane	All Text, Trial, No Word Variations		
Web of Science	English, Article		

**Table A2:** Included studies that quantified baseline physical activity (PA) or sedentary behavior (SB) indicators.

ID	First Author (Pub Yr.)—Country	Participant Characteristics		Technique (Tool–Brand Model or Type)	Baseline PA or SB Indicator_unit_ (M ± SD or Med, IQR)	n (% of N) w/Sufficient PA or SB Data; Wear (h/d)
		n (%Female); Years of Age (Age Range)	Diagnosis/-es (IQ or AB); Support Level			
1 [41]	Adolfsson (2008)—SE	32 (43.75); 35.6 ± 8.61 (26–66)	ID (NR); lower levels to more extensive	O (DO) and S (LOG–PA_score_/MIXED)	PA_score_: 1.4 ± 0.12	32 (94.1); NA
2 [42]	Ballenger (2023)—US	34 (55.9); 37.7 ± 11.6 (19–60)	DS (NR); NR	O (ACC–AG wGT3X-BT)	SB_min/d_: 395.8 ± 94.1; SB_%wear_: 48.1 ± 10.3	34 (CD); 13.8 ± 1.6
3 [43]	Barnes (2013)—US	294 (51.7); 37.8 ± 11.9 (18–65)	ID (SPMSQ NR); MMI	M1: O (ACC–AG NR) M2: S (INT–PACI/SELF)	MVPA_min/wk_: 108.6 ± 114.4 (M1); PA_activities/d_: 1.6 ± 1.5 (M2)	M1: 131 (44.6); NR M2: 294 (100.0); NA
4 [44]	Bellicha (2020)—FR	10 (100.0); 28.8, 8.8 (NR)	PWS (NR); NR	O (ACC–AG GT3X)	PA_CPM_: 211, 192; LPA_%wear_: 24, 5; MVPA_%wear_: 1.7, 4.2; MVPA^10^_min/wk_: 5, 101	7 (70.0); 12.1, NR
5 [101]	Bergström (2013)—SE	EG: 64 (57.8); 36.2 ± 10.1 (NR) CG: 66 (56.1); 39.4 ± 11.3 (NR)	ID (NR); MMI	O (PED–YM KW-LS2000) and S (LOG–AD Steps_#_/SELF)	Steps_#/d_: 8042 ± 5524 (EG), 6296 ± 4167 (CG)	EG: 46 (71.9); NR CG: 53 (80.3); NR
6 [94]	Bodde (2012)—US	42 (50); NR (19–62)	ID (NR); MMI	O (ACC–AG GT1M)	MVPA_min/wk_: 7.00 ± 21.57	25 (59.5); NR
7 [118]	Bodde (2013)—US	42 (50.0); 38.81 ± NR (19–62)	DS, ID (NR); MMI	O (ACC–AG GT1M)	MVPA_min/d_: 7.73 ± 24.21	25 (59.5); NR
8 [45]	Boonman (2019)—NL and US	27 (56.0); 29 ± 8.2 (NR)	ID (“screened for SVPR”); MMI	S (QNR–IPAQ NR/SELF)	PA_MET-min/wk_: 915, 3413	27 (CD); NA
9 [46]	Celebańska (2013)—PL	69 (39.1); 31.9 ± NR (21–54)	ID (NR); “moderate or considerable”	O (PED–YM NR) and S (LOG–AD Steps_#_/SELF)	Steps_#/d_: 6656 ± NR	69 (CD); NR
10 [47]	Chan (2012)—HK	38 (47.4); 37.68 ± 10.12 (NR)	ID (NR); MMI	O (PED–YM SW-700) and S (LOG–Steps_#_/PROXY)	Steps_#/d_: 7432 ± 4073	40 (95.0); NR
11 [48]	Chen (2018a)—US	14 (21.4); 21.54 ± 5.19 (14–29)	DS (6.32 ± 2.11 PPVT-III); NR	S (QNR–GLTEQ/PROXY)	PA_MET-min/wk_: 34.29 ± 16.96	14 (CD); NA
12 [49]	Chen (2018b)—US	18 (27.8); 21.13 ± 5.37 (14–31)	DS (NR); NR	S (QNR–GLTEQ/PROXY)	PA_MET-min/wk_: 35.78 ± 17.00	18 (CD); NA
13 [50]	Chen (2021)—US	30 (26.7); 21.26 ± 5.46 (13–31)	DS (6.04 ± 1.80 PPVT-III); NR	S (QNR–GLTEQ/PROXY)	PA_MET-min/wk_: 34.97 ± 26.28	30 (CD); NA
14 [51]	Choi (2020)—US	G1: 60 (50); 41 ± 4 (NR) G2: 20 (70) 51 ± 11 (NR)	ID (NR); MISV	O (ACC–AG wGT3X-BT)	MVPA_min/d_: 21 ± 22 (G1), 9 ± 12 (G2); LPA_min/d_: 340 ± 114 (G1), 349 ± 8 (G2)	66 (82.5); NR
15 [52]	Chow (2018)—HK	114 (37.7); 41.7 ± 9.5 (18–64)	ID (NR); MMI	O (ACC–AG wGT3X-BT)	LPA_%_: 31.1 ± 10.8; LPA_min/d_: 229.9 ± 85.3; MVPA_%_: 1.6 ± 3.4; MVPA_min/d_: 9.9 + 19.7; SB_%_: 67.3 ± 12.0; SB_min/d_: 495.4 ± 87.1	90 (78.9); 12.1 ± 1.6
16 [53]	Coats (2023)—CA	G1: 9 (100.0); 34.4 ± 13.6 (20–64) G2: 6 (0.0); 38.0 ± 14.5 (21–58)	DS, ID (NR); NR	M1: O (ACC–Polar Ignite) M2: S (LOG–Adapted Temple (2003)/SELF)	MVPA_min/wk_: 602 ± 500 (M1); LPA_min/wk_: 2342.22 ± 1281.40 (M1G1), 3496.54 ± 2468.43 (M1G2); SB_hr/wk_: 91.4 ± 36.7; SB_min/wk_: 5457.72 ± 1753.83 (M1G1), 5524.62 ± 2940.93 (M1G2)	M1: 15 (100); 21.4 ± NR M2: 15 (CD); NA
17 [102]	Curtin (2013)—US	G1: 10 (90.0); 20.5 ± 4.1(NR) G2: 11 (72.7); 20.5 ± 2.4 (NR)	G1: DS (49.1 ± 9.3 KBIT); NR G2: DS (44.8 ± 6.3 KBIT); NR	O (MS–AC MM) and S (LOG–Wear/PROXY)	MVPA_min/d_: 60.7 ± 59.6 (G1), 83.1 ± 47.7 (G2)	21 (100.0); NR
18 [54]	Dairo (2017)—UK	20 (50.0); 50 ± 16 (22–70)	ID (NR); MIPR	M1: O (ACC–AX AXS or AN GENEActiv) M2: S (QNR–IPAQ-SF/MIXED)	MVPA_min/wk_: 144.5 + 257.8 (M1), 207.1 + 240.8 (M2) MVPA + walk_min/wk_: 269.0 + 372.3 (M2)	M1: 17 (85.0); NR M2: 20 (100.0); NA
19 [55]	Dixon-Ibarra (2013)—US	G1: 45 (46.0); 32.34 ± 8.43 (20–49) G2: 31 (37.0); 57.87 ± 6.88 (50–77)	DS, ID (NR); MMI	M1: O (ACC–AG GT1M M2: O HJ 720ITC)	LPA_min/d_: 3.84 ± 1.20 (M1G1), 3.75 ± 1.42 (M1G2); MVPA_min/d_: 21.0 ± 18.6 (M1G1), 10.2 ± 13.8 (M1G2); Steps_#/d_: 6031 ± 2929 (M2G1), 4552 ± 3176 (M2G2); SB_hr/d_: 6.75 ± 1.94 (M1G1), 7.36 ± 1.77 (M1G2)	M1G1: 40 (88.9), M1G2: 28 (90.3), M2G1: 42 (93.3), M2G2: 27 (87.1); 11 ± NR
20 [126]	Dixon-Ibarra (2017)—US	18 (72.0); 59.4 ± 7.5 (NR)	ID (NR); NR	O (ACC–OM HJ-720ITC)	Steps_#/d_: 2375 ± 740	6 (33.3); 11 ± 0.76
21 [119]	Dodd (2023)—US	G1: 43 (63.0); 28.6 ± 9.4 (NR) G2: 36 (44.0); 24.5 ± 8.1 (NR)	DS (NR); NR	O (ACC–AG wGT3X-BT)	LPA_min/d_: 268.8 ± 75.0 (G1), 276.3 ± 92.6 (G2); MVPA_min/d_: 12.0 ± 11.7 (G1), 20.6 ± 28.8 (G2); SB_min/d_: 495.8 ± 143.0 (G1), 474.2 ± 113.6 (G2)	65 (82.3); NR
22 [56]	Draheim (2010)—US	52 (51.9); 42 ± 5 (35–60)	DS (NR); INLI	S (QNR–PA- NHANES III/MIXED)	MVPA_min/wk_: 188.20 ± 326.50	52 (CD); NA
23 [112]	Finlayson (2009)—UK	G1: 232 (0); NR (NR) G2: 201 (100); NR (NR)	ASD, DS, ID (NR); MIPR	S (INT–AD/SELF)	MVPA_hr/wk_: 1.8 ± NR (G1), 1.5 ± NR (G2); PA_hr/wk_: 2.9 ± NR (G1), 2.5 ± NR (G2)	433 (CD); NA
24 [114]	Finlayson (2011)—UK	62 (56.5); 37.1 ± 12.8 (18–66)	ASD, DS, ID (NR); MMI	M1: O (MS–PAL ActivPAL) M2: S (INT–AD/SELF)	Steps_#/d_: 8509 ± 4384 (M1); MPA^30^_bouts/wk_: 1.7 ± 1.7 (M1), 4.3 ± 4.5 (M2); MPA_min/wk_: 169.3 ± 197.5 (M1), 343.7 ± 278.4 (M2); SB_hr/wk_: 18.71 ± 1.88 (M1)	M1: 41 (66.1); NR M2: CD (CD); NA
25 [57]	Firkin (2023)—US	9 (55.6); 26.8 ± 5.7 (21–36)	CP, DS, ID (53.8 ± 10.0 FSIQ, 58.6 ± 11.2 VCI, 55.0 ± 7.8 PRI, 101.2 ± 34.3 ROWPVT); INLI	M1&2: O (MS–AW 4) M3: S (LOG–Mode/MIXED)	Steps_#/d_: 6015.1 ± 2169.9 (M1), 5261.5 ± 1479.8 (M2); TEE_kcal/d_: 2371.1 ± 724.9 (M1), 2243.5 ± 672.3 (M2); AEE_kcal/d_: 465.9 ± 193.7 (M1), 395.6 ± 146.0 (M2)	M1: 6 (66.7); 12.1 ± 4.7 M2: 7 (77.8); 11.3 ± 4.8 M3: 9 (100); NA
26 [58]	Fleming (2022)—US	66 (51.5); 37.77 ± 8.20 (25–55)	DS (7.67 ± 3.30 PPVT-4); MISV	O (ACC–AG GT9X) and S (LOG–Mode/MIXED)	MVPA_%_: 10.52 ± 6.79; MVPA_min/wk_: 135.00 ± 98.52; SB_%_: 43.81 ± 10.45; SB_min/d_: 534.04 ± 172.19	66 (CD); 21.1 ± 4.2
27 [59]	Frey (2004)—US	22 (50.0); 34.9 ± 9.1 (NR)	DS, ID, OSID (NR); INLI	M1: O (ACC–AG AM7164) M2: S (LOG–Mode/MIXED)	PA_CPM_: 329.0 ± 115.3 (M1); MVPA_min/d_: 19.7 ± 17.6 (M1)	M1: 22 (64.7); 13.1 ± 1.7 M2: 4 (11.8); NA
28 [60]	García-Hoyos (2017)—ES	75 (48); 33 ± 10 (18-NR)	DS (NR); NR	S (IPAQ–SF/SELF)	PA_d/wk_: 8.5 ± 4.0; PA_min/d_: 148 ± 101; PA_MET-min/wk_: 2640 ± 2314	CD (CD); NA
29 [61]	Geijer (2014)—US	G1: 33 (45); 43.7 ± 5.5 (NR) G2: 33 (45); 44.2 ± 6.0 (NR)	G1: DS (NR); NR G2: ID (NR); NR	S (QNR–PA-NHANES III/MIXED)	PA_min/wk_: 335.33 ± NR (G1), 301.15 ± NR (G2)	66 (100); NA
30 [62]	Gerald (2014)—US	G1: 10 (100); 35.1 ± 14.9 (NR) G2: 12 (0); 33.9 ± 9.3 (NR)	ID (NR); “higher functioning”	O (ACC–UCLA Wireless Community PAM)	PA_min/d_: 54.4 ± 11.6 (G1), 63.2 ± 28.3 (G2); MPA_min/d_: 6.7 ± 1.7 (G1), 13.9 ± 12.9 (G2); LPA_min/d_: 45.7 ± 11.6 (G1), 49.3 ± 23.2 (G2); SB_hr/d_: 23.1 ± 0.19 (G1), 22.9 ± 0.47 (G2)	G1: 7 (70.0); NR G2: 10 (83.3); NR
31 [63]	Ghosh (2021)—US	52 (51.9); 46 ± 14 (20–79)	CP, DS, ID (NR); MISV	O (ACC–AG wGT3X-BT)	PA_SEDBreak-#/d_: 13.5 ± 4.6; PA_SEDBreak-min/d_: 101.9 ± 61.7; SB_min/d_: 513.6 ± 139.0; SB_%wear_: 59.3 ± 11.8	52 (59.8); 14.4 ± 2.2
32 [64]	Guijarro (2008)—ES	39 (53.8); 26 ± 7 (18–45)	DS (NR); NR	S (QNR–NR/SELF)	PA_kcal/hr/wk_: 2560 ± 2144	39 (CD); NA
33 [103]	Harris (2017)—UK	G1: 26 (69.2); 40.6 ± 15.0 (NR) G2: 24 (58.3); 43.6 ± 14.0 (NR)	DS, FXS, ID (NR); MIPR	M1: O (ACC–AG GT3X+) M2: S (IPAQ–SF/MIXED)	MVPA_%/d_: 4.5 ± 2.7 (M1G1), 4.7 ± 3.8 (M1G2); LPA_%/d_: 21.8 ± 6.2 (M1G1), 22.3 ± 8.0 (M1G2); SB_%/d_: 73.7 ± 7.6 (M1G1), 73.0 ± 9.7 (M1G2)	M1G1: 25 (96.2); NR M1G2: 22 (91.7); NR M2: 0 (0); NA
34 [120]	Harris (2019a)—UK	143 (51.7); 45.3 ± 13.6 (18-NR)	ID (NR); MIPR	O (ACC–AG GT3X+)	PA_SEDBreak-#/d_: 7.0, 7.0; PA_SEDBreak-min/bout_: 43.2, 30.5; SB_hr/d_: 8.1, 4; SB_min/d_: 491.3, 239; SB_%wear_: 73.0 ± 10.4	143 (94.1); 11.3 ± NR
35 [127]	Harris (2019b)—UK	G1: 26 (69.2); 40.6 ± 15 (NR) G2: 24 (58.3); 43.6 ± 14 (NR)	ID (NR); MIPR	M1: O (ACC–AG GT3X+) M2: S (IPAQ–SF/NR)	PA_min/d_: 176.8 ± 53.3 (M1G1), 191.2 ± 85.1 (M1G2); SB_min/d_: 501.1 ± 125.9 (M1G1), 522.3 ± 165.3 (M1G2)	M1G1: 25 (96.2); NR M1G2: 22 (91.7); NR M2: 0 (0); NA
36 [65]	Hilgenkamp (2012a)—NL	257 (48.2); ~60.0 ± NR (50–89)	DS, ID (NR); BOSV	O (PED–NL 1000) and S (LOG–Steps_#_/PROXY)	Steps_#/d_: 6601 ± 3610	257 (24.5); NR
37 [66]	Hilgenkamp (2012b)—NL	268 (48.5); ~59.9 ± NR (50–84)	DS, ID (NR); BOSV	O (PED–NL 1000) and S (LOG–Steps_#_/PROXY)	Steps_#/2-Mons_: 6946 ± 4599; Steps_#/2-Tues_: 7050 ± 4532; Steps_#/2-Weds_: 6977 ± 4342; Steps_#/2-Thus_: 7099 ± 4561; Steps_#/2-Fri_: 6937 ± 4227; Steps_#/2-Sat_: 6098 ± 4026; Steps_#/2-Sun_: 5293 ± 4261	135 (50); NR
38 [104]	Højberg (2022)—DK	EG: 52 (53.8); 26.7 ± 1.2 (NR) CG: 14 (35.7); 29.1 ± 1.7 (NR)	ASD, CP, DS, FXS, ID, OSID (NR); MMI	O (ACC–Axivity AX3)	PA_min/wk_: 407 ± NR (EG), NR (CG); Steps_#/hr/wkd_: 738 ± 67 (EG), NR (CG); Steps_#/hr/wknd_: 564 ± 58 (EG), NR (CG)	CD (CD); NR
39 [121]	Hsieh (2017)—US	1618 (44.8); 37.67 ± 14.39 (18–86)	ASD, CP, DS, ID (NR); BOPR	S (QNR–NR/MIXED)	SB_TVhr/d_: 3.42 ± 2.13	1618 (57.0); NA
40 [67]	Hsu (2021)—TW	60 (45); 39.19 ± 11.70 (19.20–70.20)	ID (NR); MMI	O (ACC–AG GT3X)	Steps_#/d_: 6486 ± 2935; MVPA_min/d_: 16.67 ± 13.84; LPA_min/d_: 275.25 ± 76.86; SB_min/d_: 517.69 ± 103.19	60 (100); NR
41 [95]	Jo (2018)—KR	EG: 10 (30); 30.60 ± 11.00 (18-NR) CG: 10 (70); 31.80 ± 11.32 (18-NR)	ID (NR); NR	O (ACC–AG GT3X+)	Steps_#/d_: 6670 ± 2090 (EG), 5300 ± 1260 (CG); PA_kcal/d_: 78.37 ± 37.18 (EG), 67.86 ± 31.53 (CG); MVPA_min/d_: 43.29 ± 21.59 (EG), 29.83 ± 14.13 (CG); SB_hr/d_: 14.77 ± 1.62 (EG), 13.66 ± 2.32 (CG)	EG: 8 (80.0); NR CG: 6 (60.0); NR
42 [108]	Lante (2011)—US	2 (50); P1: 21y P2: 22y	ID (NR); MI	O (ACC–AG GT1M)	LPA_min/wkd_: 58.44 ± 1.18 (P1), 59.32 ± 0.57 (P2); LPA_min/wknd_: 59.60 ± 0.24 (P1), 58.98 ± 1.59 (P2); LPA_%wake/wkd_: 97.40 (P1), 98.88 (P2); LPA_%wake/wknd_: 99.33 (P1), 98.30 (P2); MVPA_min/wkd_: 1.56 ± 1.18 (P1), 0.67 ± 0.57 (P2); MVPA_min/wknd_: 0.40 ± 0.24 (P1), 1.02 ± 1.59 (P2); MVPA_%wake/wkd_: 2.60 (P1), 1.22 (P2); MVPA_%wake/wknd_: 0.67 (P1), 1.70 (P2); Steps_#/wake/wkd_: 386.04 ± 128.14 (P1), 434.24 ± 189.92 (P2); Steps_#/wake/wknd_: 208.32 ± 42.88 (P1), 297.70 ± 241.72 (P2)	2 (100); NR
43 [109]	Li (2019)—US	4 (50); 23 ± 1.41 (21–25)	ASD, ID (NR); NR	O (ACC–FB ZIP)	Steps_#/d_: 6935.8 ± 857.6	4 (100); NR
44 [128]	Maine (2019)—UK	48 (37.5); 20.9 ± 5.02 (18–39)	ID (NR); MI	M1: O (PED–YM SW-200) and S (LOG–Steps_#_/SELF) M2: S (QNR–IPAQ NR/SELF)	Steps_#/d_: 4485.7 ± 3211 (M1); PA_MET_: 598, NR (M2); PA_MET-transport_: 165, NR (M2); PA_MET-household_: 150.75, NR (M2); PA_MET-recreational_: 224.75, NR (M2)	M1: 48 (CD); NR M2: 48 (CD); NA
45 [68]	Martin (2011)—UK	28 (35.9); NR ± NR (25–54)	ID (NR); NR	S (QNR–IPAQ SF/PROXY)	PA_MET-min/14d_: 1066, 1670.25; MPA_MET-min/14d_: 240, 720; VPA_MET-min/14d_: 0, 540	28 (13.3); NA
46 [122]	Matthews (2011)—UK	45 (62.2); 48.3 ± 12.01 (23–72)	DS, ID (NR); MISV	M1: O (ACC–AG GT1M) and S (LOG–Wear/MIXED) M2: S (IPAQ–SF/MIXED)	MPA_min/d_: 12.82 ± 15.98 (M1), 7.17 ± 12.38 (M2); SB_hr/d_: 10.17 ± 2.06 (M1), 9.36 ± 3.21 (M2)	M1 + M2: 45 (83.3); NR
47 [129]	Matthews (2016)—UK	54 (46); 45 ± 14 (NR)	ID (NR); MISV	O (PED–NR) and S (LOG–Steps_#_/MIXED)	Steps_#/d_: 4744 ± 2076	54 (CD); NR
48 [105]	McDermott (2012)—US	443 (50.3); 38.8 ± NR (19–70) EG: 216 CG: 216	ID (NR); MMI	O (ACC–AG NR)	MVPA_%wear_: 2.0 ± NR; LPA_%wear_: 10.6 ± NR; SB_%wear_: 87.4 ± NR; MVPA_min/wear-min_: 0.024 ± 0.025 (EG), 0.02 ± 0.0301 (CG)	401 (90.5); 9.7 ± NR EG: 61 (28.2); NR CG: 57 (26.4); NR
49 [69]	McKeon (2013)—IE	17 (0); 42 ± NR (19–59)	ID (NR); MIPR	M1: O (ACC–BM SWA) M2: S (QNR–IPAQ NR/SELF)	Steps_#/d_: 5308 ± 5502 (M1); TEE_kcal/d_: 1970 ± 598 (M1); AEE_kcal/d_: 377 ± 425 (M1); PA_h/d_: 1.24 ± 1.01 (M1); PA_kcal/kg/h_: 1.40 ± 0.24 (M1); MPA_h/d_: 1.21 ± 1.00 (M1); VPA_h/d_: 0.03 ± 0.04 (M1); SB_h/d_: 15.00 ± 6.00 (M1)	M1: 17 (CD); 16 ± 6 M2: 17 (CD); NA
50 [96]	Melo (2021)—PT	15 (40); 30.1 ± 7.5 (NR)	ASD, DS, ID (NR); MMI	O (ACC–AG GT1M)	MVPA_min/wk_: 270.2 ± 150.5	15 (CD); NR
51 [124]	Melville (2011)—UK	54 (59.3); 48.3 ± 12.01 (23–71)	DS, ID (NR); MIPR	M1: O (ACC–AG GT1M) and S (LOG–Wear/SELF) M2: S (QNR–IPAQ-SF/MIXED)	PA_walk-min/wk_: 48.7 ± 54.3 (M2); MVPA_min/d_: 13.1 ± 16.2 (M1); MVPA_min/wk_: 55.4 ± 89.0 (M2); MVPA_%_: 2.0 ± 2.7 (M1); LPA_min/d_: 69.9 ± 43.83 (M1); LPA_%_: 10.1 ± 6.0 (M1); SB_min/d_: 612 ± 121.75 (M1), 557.4 ± 189.4 (M2); SB_%_: 87.9 ± 7.7 (M1)	M1: 54 (100.0); NR M2: 47 (87.0); NA
52 [106]	Melville (2015)—UK	EG: 54 (46.3); 44.9 ± 13.5 (NR) CG: 48 (41.7); 47.7 ± 12.3	ID (NR); MISV	M1: O (ACC–AG GT3X) and S (LOG–Wear/MIXED) M2: S (QNR–IPAQ-SF/MIXED)	PA_%/d_: 35.8 ± 10.4 (M1EG), 33.1 ± 11.3 (M1CG); PA_MET-min/wk_:1367.6 ± 1629.9 (M2EG), 1150.1 ± 1059.9 (M2CG); MVPA_%/d_: 3.2 ± 2.7 (M1EG), 3.3 ± 2.9 (M1CG); SB_%/d_: 64.2 ± 10.5 (M1EG), 66.9 ± 11.3 (M1CG); Steps_#/d_: 4744 ± 2076 (M1EG), 4818 ± 2784 (M1CG)	M1EG: 54 (100); NR M1CG: 48 (100); NR M2EG: 53 (98.1); NA M2CG: 40 (83.3) NA
53 [97]	Merzbach (2023)—CA, DE, FI, IE, MM, NZ, TH, UK, US, ZA	83 (48.2); 27.1 ± 8.0 (NR)	DS (Raven’s APM–Set 1: 4.9 ± 2.8); NR	M1: O (ACC–FB Inspire 2) M2: S (QNR–NR/SELF)	PA_walkbike-min/wk_: 186.4 ± 127.4 (M2, *n* = 41); MPA_min/wk_: 404.6 ± 866.5 (M2, *n* = 52); VPA_min/wk_: 199.3 ± 308.6 (M2, *n* = 14); SB_sit-min/d_: 425.8 ± 214.8 (M2, *n* = 83)	M1: NR (NR); NR M2: 14–83 (16.7–100); NA
54 [70]	Mikulovic (2014a)—FR	570 (41.0); 38.1 ± 10.3 (19–59)	ID (NR); NR	S (QNR–AD NA/SELF)	PA_worksport-h/wk_: 4, 5; SB_h/wk_: 18, 16	570 (82.5); NA
55 [71]	Mikulovic (2014b)—FR	G1: 119 (47.1); 41 ± 10.10 (NR) G2: 171 (47.4); 39 ± 10.79 (NR) G3: 100 (30); 41 ± 9.82 (NR) G4: 119 (37.8); 38 ± 9.48 (NR)	ID (NR); NR	S (QNR–AD NA/SELF)	PA_min/wk_: 390 ± 450 (G1), 256 ± 233 (G2), 386 ± 282 (G3), 276 ± 270 (G4); SB_h/wk_: 20.25 ± 12.25 (G1), 17.75 ± 12.76 (G2), 23.82 ± 14.89 (G3), 27.20 ± 17.46 (G4); SB_TV-min/d_: 218 ± 131 (G1), 180 ± 125 (G2), 220 ± 143 (G3), 225 ± 130 (G4); SB_CP + VG-min/d_: 131 ± 178 (G1), 129 ± 71 (G2), 163 ± 116 (G3), 172 ± 130 (G4)	509 (CD); NA
56 [72]	Moss (2018)—ZA	56 (50); 39.6 ± 9.1 (NR)	ID (NR); NR	M1: O (ACC–CNT AH) M2: S (QNR–IPAQ-SF/PROXY)	PA_min/wk_: 225.6 ± 92.0 (M1), 177.1 ± 309.2 (M2); PAL_score_: 1.39 ± 0.15 (M1); TEE_kcal/d_: 1974 ± 385 (M1); AEE_kcal/d_: 3660 ± 215 (M1)	M1: 56 (93.3); 20 ± NR M2: 56 (93.3); NA
57 [110]	Nastasi (2020)—US	4 (50); 37 ± 15.17 (23–55)	ASD, ID (NR); BOMI	O (ACC–FB FLEX)	Steps_#/d_: 4650.3 ± 1426.7	4 (80); NR
58 [73]	Nordstrøm (2013)—NO	87 (62.1); 28.5 ± 7.5 (16–45)	DS, PWS, OSID (NR); NR	M1: O (ACC–AG GT3X+) M2: S (LOG–AD/NR)	PA_CPM_: 294 ± 121.1; Steps_#/d_: 6712 ± 3167; MVPA_min/d_: 27.1 ± 21.1; MVPA^10^_min/d_: 11.1 ± 13.7; LPA_min/d_: 212 ± 61.4; PA_lifestyle-min/d_: 71.2 ± 33.4; SB_min/d_: 522 ± 80.3	M1: 83 (95.4); 13.9 ± 1.4 M2: CD (CD); NA
59 [113]	Oppewal (2014)—NL	G1: 539 (48.4); 60.7 ± 7.8 (NR) G2: 142 (52.1); 61.5 ± 6.8 (NR) G3: 43 (58.1); 63.2 ± 8.0 (NR)	DS, ID (NR); BOPR	O (PED–NL 1000) and S (LOG–CD)	Steps_#/d_: 6830.1 ± 3772.9 (G1), 8003.5 ± 4115.0 (G2), 5682.7 ± 2207.8 (G3)	191 (26.4); NR
60 [74]	Oreskovic (2020)—US	52 (53.8); 35.1 ± 10.8 (18–60)	DS (NR); NR	O (ACC–AG wGT3X-BT or GT3X)	Steps_#/d_: 3194 ± 1988; MVPA_min/d_: 11.7 ± 22.4; MPA_min/d_: 10.1 ± 13.5; VPA_min/d_: 1.7 ± 9.8; LPA_min/d_: 161.0 ± 85.5; SB_min/d_: 412.7 ± 216.6	52 (82.5); 9.6 ± NR
61 [130]	Overwijk (2022)—NL	G1: 21 (NR); NR (NR) G2: 3 (NR); NR (NR)	ID (NR); MOPR	G1: O (ACC–AG wGT3X-BT) G2: O (ACC–PR ActiWatch)	PA_%_: 41.33 ± 29.54 (G2); VVPA_%_: 0.01 ± 0.04 (G1); VPA_%_: 0.17 ± 0.40 (G1); MPA_%_: 2.46 ± 2.70 (G1); LPA_%_: 33.25 ± 14.94 (G1); SB_%_: 64.11 ± 15.53 (G1), 58.67 ± 29.54 (G2)	G1: 14 (58.3); NR G2: 3 (100); NR
62 [75]	Oviedo (2017)—ES	92 (42.39); 44 ± 12 (NR)	ASD, CP, DS, ID, MC, OSID (NR); MISV	O (ACC–AG GT3X)	Steps_#/d_: 6192 ± 2814; PA_CPM_: 251.9 ± 123.2; MVPA_min/d_: 30.8 ± 22.5; LPA_min/d_: 128.3 ± 47.0; SB_min/d_: 612.9 ± 80.1	84 (91.3); 12.9 ± 1.3
63 [76]	Oviedo (2019)—ES	G1: 37 (40.5); 41 ± 11 (NR) G2: 29 (41.4); 46 ± 12 (NR)	ID (NR); MMI	O (ACC–AG GT3X)	PA_CPM_: 306.86 ± 85.71 (G1), 236.54 ± 107.90 (G2); MVPA_min/d_: 38.72 ± 26.64 (G1), 25.95 ± 20.58 (G2); MPA_min/d_: 37.48 ± 26.29 (G1), 25.26 ± 19.87 (G2); VPA_min/d_: 1.24 ± 0.99 (G1), 0.67 ± 0.45 (G2); LPA_min/d_: 614.98 ± 106.77 (G1), 615.04 ± 80.57 (G2); PA_SEDBreak#/SEDhr_: 11.64 ± 1.80 (G1), 12.37 ± 2.15 (G2); SB^1^_min/d_: 123.34 ± 20.13 (G1), 124.69 ± 20.79 (G2)	G1: 35 (94.6); 12.9 ± 1.9 G2: 28 (96.6); 13.0 ± 1.2
64 [99]	Pérez-Cruzado (2016)—ES	40 (15); 35.86 ± 9.93 (NR)	ID (NR); MI	S (QNR–IPAQ NR/SELF)	PA_walk-MET-min/7d_: 672.62 ± NR; VPA_MET-min/7d_: 5372.57 ± NR; MPA_MET-min/7d_: 645.71 ± NR	CD (CD); NA
65 [77]	Pérez-Cruzado (2018)—ES	33 (27.3); 35.86 ± 9.92 (18–60)	ID (NR); MI	M1: O (ACC–AG GT3X) M2: S (QNR–IPAQ SF/SELF)	PA_min/wk_: 582.90 ± 771.24 (M1); PA_MET-min/7d_: 7014.78 ± 7509.26 (M2); MPA_min/wk_: 140.48 ± 199.69 (M1); MPA_MET-min/7d_: 541.21 ± 624.41 (M2); VPA_min/wk_: 17.66 ± 56.84 (M1); VPA_MET-min/7d_: 4854.54 ± 7357.96 (M2); LPA_min/wk_:424.76 ± 756.07 (M1); LPA_MET-min/7d_: 1619.03 ± 2043.86 (M2)	M1: 33 (100); NR M2: CD (CD); NA
66 [78]	Peterson (2008)—US	131 (51.9); 37.2 ± 11.6 (18–60)	DS, ID (NR); MMI	O (ACC–OM HJ-700IT)	Steps_#/d_: 6621 ± 3366	131 (86.8); 14.5 ± 2.2
67 [79]	Phillips (2011)—UK	G1: 38 (100); NR (16–34) G2: 14 (100); NR (35–44) G3: 9 (100); NR (45–54) G4: 13 (100); NR (55–64) G5: 37 (0); NR (16–34) G6: 17 (0); NR (35–44) G7: 9 (0); NR (45–54) G8: 8 (0); NR (55–64)	ASD, DS, ID, OSID (NR Leicester- shire ID tool); MISV	M1: O (ACC–AG GT1M) M2: S (LOG–Wear/MIXED)	PA_CPM_: 577.0 ± 138.7 (G1), 556.3 ± 87.7 (G2), 577.7 ± 485.0 (G3), 485.0 ± 170.7 (G4), 694.7 ± 184.2 (G5), 739.0 ± 418.3 (G6), 602.0 ± 182.7 (G7), 585.0 ± 264.8 (G8); Steps_#/d_: 5648 ± 1831 (G1), 6274 ± 2021 (G2), 6751 ± 2090 (G3), 4649 ± 2126 (G4), 6558 ± 2493 (G5), 7376 ± 4199 (G6), 6682 ± 2831 (G7), 7723 ± 5168 (G8); MVPA_min/d_: 32.1 ± 13.5 (G1), 31.8 ± 9.9 (G2), 35.4 ± 16.5 (G3), 21.5 ± 14.7 (G4), 42.2 ± 19.1 (G5), 45.2 ± 34.1 (G6), 31.3 ± 20.1 (G7), 39.0 ± 34.2 (G8); MVPA^10^_min/d_: 0.4 ± 1.3 (G1), 0.8 ± 2.9 (G2), 0.0 ± 0.0 (G3), 0.0 ± 0.0 (G4), 0.9 ± 1.9 (G5), 1.6 ± 3.2 (G6), 0.0 ± 0.0 (G7), 1.8 ± 3.4 (G8); MPA_min/d_: 30.0 ± 12.3 (G1), 30.8 ± 9.9 (G2), 33.5 ± 15.3 (G3), 20.7 ± 14.8 (G4), 39.6 ± 17.3 (G5), 42.8 ± 33.0 (G6), 29.5 ± 18.6 (G7), 38.3 ± 33.9 (G8); VPA_min/d_: 2.1 ± 3.3 (G1), 1.0 ± 0.4 (G2), 2.0 ± 2.1 (G3), 0.8 ± 0.4 (G4), 2.6 ± 2.8 (G5), 2.3 ± 3.2 (G6), 1.8 ± 2.7 (G7), 0.7 ± 0.5 (G8); LPA_min/d_: 113.6 ± 27.8 (G1), 139.9 ± 38.2 (G2), 143.2 ± 54.0 (G3), 104.9 ± 33.2 (G4), 112.9 ± 30.5 (G5), 121.4 ± 41.5 (G6), 131.3 ± 36.2 (G7), 119.9 ± 52.6 (G8); SB_min/d_: 644.2 ± 40.6 (G1), 560.4 ± 84.7 (G2), 576.0 ± 49.5 (G3), 605.1 ± 86.0 (G4), 604.0 ± 65.2 (G5), 586.7 ± 143.8 (G6), 603.6 ± 96.7 (G7), 648.2 ± 95.1 (G8)	M1: 152 (88.9); ID w/out DS (*n* = 73): 12.5 ± 1.2, DS (*n* = 79): 12.7 ± 1.2
68 [115]	Powers (2021)—US	12 (58.33); 34.67 ± 7.75 (NR)	ID (NR); MMI	S (QNR–IPAQ SF/MIXED)	SB_h/wkd_: 9.13 ± 4.6; SB_h/wknd_: 10.71 ± 4.9	12 (100.0); NA
69 [107]	Ptomey (2018)—US	149 (57.0); NR ± NR (NR)	ASD, DS, ID (NR); MMI	O (ACC–AG GT1X)	MVPA_min/d_: ~15 ± NR	98 (65.8); NR
70 [123]	Ptomey (2020)—US	DS: 21 (66.7); 36.5 ± 9.6 (NR) ID: 103 (54.4); 36.5 ± 12.5 (NR)	DS, ID (NR); MMI	O (ACC–AG GT1X)	MVPA_%/wear_: 0.8 ± 0.9 (DS), 1.5 ± 3.8 (ID); LPA_%/wear_: 34.6 ± 13.2 (DS) ID: 34.3 ± 29.3 (ID); SB_%/wear_: 64.0 ± 13.8 (DS), 63.6 ± 30.8 (ID)	124 (CD); 11 ± NR
71 [80]	Rodrigues (2019)—PT	78 (47.4); 30.18 ± 8.55 (18–53)	ID (NR); MMI	O (PED–YM SW-700) and S (LOG–Steps_#_/SELF)	Steps_#/d_: 4865.21 ± 1611.04 (M1), 3739.22 ± 1243.34 (M2)	78 (100); NR
72 [131]	Salomon (2023)—AU	6 (33.3); 46.0 ± 13.0 (28–62)	ID (NR); NR	M1: O (ACC–AG GT3X) M2: S (QNR–IPAQ-PR PROXY)	MVPA_min/d_: 25.56 ± 12.98 (M1); MPA_min/d_: 24.96 ± 13.38 (M1); VPA_min/d_: 0.60 ± 0.97 (M1); LPA_min/d_: 108.02 ± 78.72 (M1); SB_min/d_: 643.94 ± 198.07 (M1)	M1: 5 (83.3); NR M2: 2 (33.3); NA
73 [81]	Shields (2018)—AU	12 (25); 25.3 ± 10.1 (NR)	DS (NR); MMI	O (ACC–AG GT3X)	PA_CPM_: 294.4, 151.4; MVPA_min/d_: 27.2, 39.8; MVPA^10^_min/d_: 11.6, 24.2; MPA_min/d_: 22.3, 27.2; MPA^10^_min/d_: 8.1, 21.0; VPA_min/d_: 5.0, 12.2; VPA^10^_min/d_: 0.0, 2.0	12 (80.0); 12.5 ± 1.7
74 [82]	Soler Marín (2011)—ES	38 (39.5); NR ± NR (16–38)	DS (NR); NR	S (QNR–NR PROXY)	PAL_score_: 1.3 ± NR	38 (CD); NA
75 [98]	Spanos (2016)—UK	28 (64); NR ± NR (NR)	ID (NR); MISV	M1: O (ACC–AG GT1M) M2: S (QNR–IPAQ SF/SELF)	PA_walk-min/1d_: 60.3 ± 55.0 (M2); MVPA_min/d_: 19.3 ± 17.3 (M1); MVPA_%_: 3.2 ± 3.6 (M1); LPA_min/d_: 82.6 ± 38.2 (M1); LPA_%_: 12.6 ± 6.2 (M1); SB_min/d_: 576.5 ± 145.9 (M1); SB_%_: 84.2 ± 8.7 (M1)	M1: 18 (64.3); NR M2: 28 (100); NA
76 [83]	Stanish (2004)—CA	G1: 8 (100); NR (NR) G2: 4 (100); NR (NR) G3: 3 (0); NR (NR) G4: 5 (0); NR (NR)	DS, ID (NR); MI G1: ID G2: DS G3: ID G3: DS	O (PED–YM SW-500) and S (LOG–Steps_#_/PROXY)	Steps_#/d_: 11,809.4 ± 4652.4 (G1), 8815.6 ± 4094.1 (G2), 11,885.3 ± 5645.9 (G3), 5449.8 ± 2316.3 (G4)	20 (CD); NR
77 [85]	Stanish (2005a)—CA	G1: 38 (100); 39.7 ± 9.5 (NR) G2: 65 (0); 35.9 ± 11.2 (NR)	DS, ID (NR); MMI	O (PED–YM SW-500 or SW-700) and S (LOG–Steps_#_/SELF)	Steps_#/wk_: 53,312 ± 26,629 (G1), 55,703 ± 27,218 (G2)	103 (CD); NR
78 [84]	Stanish (2005b)—CA	103 (36.9); 37.3 ± 10.7 (19–65)	DS, ID (NR); MMI	M1: O (PED–YM SW-500 or SW-700) and S (LOG–Steps_#_/MIXED) M2: S (QNR–PA section of the NHANES III/MIXED)	Steps_#/wk_: 54,821 ± 26,896 (M1); PA_walk-min/wk_: 55.4 ± 98.5 (M2); MVPA_min/wk_: 209.2 ± 232.8 (M2)	103 (CD); NR
79 [86]	Stanish (2007)—US	G1: 26 (42.3); 37.7 ± 9.1 (NR) G2: 30 (36.7); 37.1 ± 22.0 (NR) G3: 25 (32.0); 37.8 ± 12.3 (NR) G4: 22 (36.4); 36.4 ± 10.9 (NR)	DS, ID (NR); NR	O (PED–YM SW-500 or SW-700) and S (LOG–Steps_#_/MIXED)	Steps_#/d_: 3712 ± 936 (G1), 6285 ± 746 (G2), 8592 ± 680 (G3), 13,945 ± 2257 (G4)	103 (CD); NR
80 [87]	Sundahl (2016)—SE	52 (51.9); 18.2 ± 1.2 (16.0–20.0)	ID (NR); MMI	O (PED–YM KW-LS2000 or KW-LS7000) and S (LOG–Steps_#_/MIXED)	Steps_#/5d_: 44,890 ± 20,342	49 (94.2); NR
81 [111]	Temple (2000)—AU	6 (50); 35.5 ± 3.58 (19–45)	ID (NR); MMI	M1: O (ACC–MDFN Caltrac) M2: O (DO) and S (LOG–BCH NA/PROXY)	MVPA_min/d_: 38.6 ± NR (M1); LPA_C4-min/d_: 3 ± NR (M2); LPA_C3-stand-hr/d_: 3 ± NR (M2); SB_sitting-C2-hr/d_: 6 ± NR (M2); SB_lying-C1-hr/d_: 10 ± NR (M2)	M1: 6 (100); NR M2: 6 (100); NA
82 [88]	Temple (2003)—AU	37 (48.6); NR ± NR (NR)	ID (NR); MMI	M1: O (ACC–MDFN Caltrac) M2: O (DO) and S (LOG–BCH NA/PROXY)	TEE_#/d_: 2524.0 ± 502.3 (M1), 2262.9 ± 519.5 (M2); MVPA_min/d_: 4.3 ± 13.2; VPA_C9min/d_: 0.0 ± 0.0 (M2); MPA_C8min/d_: 4.3 ± 13.2(M2); MPA_C7min/d_: 0.0 ± 0.0 (M2); LPA_C6-min/d_: 7.3 ± 12.3 (M2); LPA_C5-min/d_: 68.0 ± 63.9 (M2); LPA_C4-min/d_: 56.2 ± 63.6 (M2); LPA_C3-stand-hr/d_: 96.6 ± 103.3 (M2); SB_sitting-C2-hr/d_: 623.5 ± 161.7 (M2); SB_lying-C1-min/d_: 582.8 ± 122.0 (M2)	M1: 37 (94.8); NR M2: 37 (94.8); NA
83 [89]	Temple (2007)—CA	37 (51.4); 32.6 ± 9.4 (18–52)	ID (NR); INET	O (PED–YM NL SW-700) and S (LOG–Steps_#_/MIXED)	Steps_#/d_: 8100.5 ± 3735.4	37 (CD); NR
84 [116]	Temple (2009)—CA	13 (53.8); 34 ± 8 (18–46)	ID (NR); NR	O (PED–YM NL SW-700) and S (LOG–Steps_#_/SELF)	Steps_#/d_: 11,928 ± 2421	13 (100); NR
85 [90]	Tomaszewski (2022)—US	38 (28.9); 25.79 ± 8.60 (18–55)	ASD, ID (60.2 ± 10.3 Leiter-3); MI	O (ACC–FB FLEX)	Steps_#/wk_: 51,759 ± 24,729; Steps_#/d_: 7394 ± 3533	38 (82.6); NR
86 [117]	Vlot-van Anrooij (2018)—NL	40 (55.0); 37.0 ± 15.5 (18–76)	ID (NR); MI	S (QNR SBQ ID/SELF)	SB_h/d_: 10, 9.61	16 (40.0); NA
87 [125]	Walsh (2018)—IE	146 (42.8); 33.01 ± 11.09 (16–64)	ID (NR); MISV	M1: O (ACC–AG GT3X) M2: S (QNR–SLAN/SELF)	MVPA_min/d_: 50.9 ± 33.3 (M1), 22.3 ± 30.6 (M2); MPA_min/d_: 18.9 ± 32.3 (M2); VPA_min/d_: 5.5 ± 10.2 (M2); LPA_min/d_: 227.2 ± 87.0 (M1), 25.6 ± 38.4 (M2); SB_min/d_: 679.5 ± 182.1 (M1)	M1: 80 (54.8); NR M2: 136 (93.2); NA
88 [91]	Woods (2018)—US	19 (57.9); 34.5 ± 4.3 (18–62)	ID (NR); NR	O (ACC–PAL activPAL NR) and S (LOG–Wear/PROXY)	Steps_#/d_: 7631.7 ± 1171	19 (86.4); NR
89 [92]	Xu and Choi (2020)—US	58 (50.0); 44 ± 14 (20–74)	CP, DS, ID (NR);	O (ACC–AG wGT3X-BT) and S (LOG–Wear/PROXY)	MPA_min/d_: 18.8 ± 21.0; VPA_min/d_: 0.2 ± 0.6; LPA_min/d_: 351.8 ± 105.1; SB_min/d_: 492.8 ± 130.1	58 (72.5); NR
90 [100]	Yan (2015)—US	22 (40.9); 26.7 ± NR (22–34)	ID (NR); MMI	O (PED–YM SW-700) and S (LOG–Steps_#_/PROXY)	Steps_#/h_: 462.00 ± 290.05	22 (CD); NR
91 [93]	Zwack (2022)—AU	39 (25.6); 31.5 ± 6.6 (NR)	ASD, CP, DS, ID (NR); MIPR	S (INT–AD 7-d Recall/MIXED)	PA_min/wk_: 172.2 ± 148.9; VPA_min/wk_: 21.5 ± 70.9; MPA_min/wk_: 73.6 ± 118.6; LPA_min/wk_: 77.8 ± 163.5	39 (100); NA

Notes. NR: not reported. CD: cannot determine. *Country abbreviations* follow the ISO alpha-2 coding system. ACC: accelerometer; AD: author derived; AEE: active energy expenditure; ASD: autism spectrum disorder; BOMI: borderline-to-mild impairment; BOPR: borderline-to-profound impairment; BOSV: borderline-to-severe impairment; CPM: counts per minute; CP: cerebral palsy; DS: Down syndrome; FSIQ: Full-Scale Intelligence Quotient; INET: intermittent-to-extensive support; INLI: intermittent-to-limited support; INT: interview; KBIT: Kaufman Brief Intelligence Test; LOG: log or diary; LPA: light-intensity physical activity; MC: microcephaly; MET: metabolic-equivalent-of-task; MI: mild impairment; MIPR: mild-to-profound impairment; MISV: mild-to-severe impairment; MMI: mild-to-moderate impairment; MOPR: moderate-to-profound impairment; MPA: moderate-intensity physical activity; MVPA: moderate-to-vigorous-intensity physical activity; O: objective technique; OSID: other rare syndrome related to intellectual disability not including Down syndrome or Prader–Willi syndrome; PAL: physical activity level; PED: pedometer; PPVT-III: Peabody Picture Vocabulary Test-Third Edition; PWS: Prader–Willi syndrome; QNR: questionnaire; S: subjective technique; SED: sedentary; TEE: total energy expenditure; #: count.
