# Supplementary material for: Quantifying Physical Activity and Sedentary Behavior in Adults with Intellectual Disability: A Scoping Review of Assessment Methodologies
Source: Healthcare (Basel). 2024 Sep 24;12(19):1912. doi: 10.3390/healthcare12191912 (PMC11476182; doi:10.3390/healthcare12191912)
Supplement: Supplementary file 1 [file healthcare-12-01912-s001.zip › healthcare-3176219-supplementary.pdf]

Supplemental Materials

Quantifying Physical Activity and Sedentary Behavior in Adults with Intellectual Disability: A Scoping Review of Assessment Methodologies

Cora J. Firkin [1,*], Iva Obrusnikova [1] and Laura C. Koch [2]

[1] Department of Health and Nutrition Sciences, University of Delaware, Newark, DE, USA
[2] Temerty Faculty of Medicine, University of Toronto, Toronto, ON, Canada
* Correspondence: cjfirkin@udel.edu

Table S1. Wearable specifications to quantify physical activity and sedentary behavior in adults w/ intellectual disability, by the manufacturer.

Model (Release Yr); Type (Axes, if applicable)	ID	Placement (Apparatus)	f_s (Hz), Epoch length (s)	Non-wear-time definition (Allowance, if applicable)	Total days; Minimum: h/d, d/wk (wknd d)	Cut-point Parameter; Step-count Index	Software: Processing; Analysis	Removal Instruction
ActivInsights Ltd (Kimbolton, UK)								
GENEActiv (CD); ACC (3)	18	Wrist (Wrist Band)	1000[†], NR	NR	7; NR, 7d (2d)	Esliger (2011) [32]; NA	GeneActiv v.NR; NR	Sleep
ActiGraph LLC (Pensacola, US)								
AM7164 (1993); ACC (1)	3	Waist (NR)	10[†], 10[¥]	NR	7; 8h, 4d (1d)	Mod-Freedson (1998) [31]; NA	ActiLife v.NR; SAS v.9.2	Sleep
	27	R Hip (Pouch on elastic belt)	10[†], 60	Log or diary entries	7; 10h, 5d (1d)	Freedson (1998) [31]; NA	NR; SPSS v.NR	Bathe, water activity
GT1M (2005-2008[d]); ACC (1-2[d])	6	NR (Belt/waist band)	30[†], 5[¥]	NR	7; 8h, 3d (NR)	Freedson (1998) [31]; NA	SOLAS v.NR; NR	Bathe, sleep, swim
	7	Waist (Belt/waist band)	30[†], 5[¥]	NR	7; 8h, 3d (NR)	NR	SOLAS v.NR; SPSS v.17.0	Bathe, sleep, swim
	19	Waistline over hip (NR)	30[†], 60	≥60 consec min of 0 CPM (1-2 min of 1-100)	7; NA, 4d (NR)	Troiano (2008) [133]; NA	ActiLife v.5, Excel; STATA v.11.2	Sleep
	42	Waist (Elastic belt)	30[†], 5[¥]	NR	28; each NR	Freedson (1998) [31]; NA	NR; NR	Sleep, water activity
	46	NR (NR)	30[†], 5[¥]	Diary entries	7; each NR	Freedson (1998) [31]; NA	ActiLife v.3.3.0; SPSS v.15.0	Bathe, sleep
	50	R Hip near the iliac crest (NR)	30[†], 15 reintegrate to 60	≥60 consec min of 0 CPM	7; 10hr, 3d (1d)	Troiano (2008) [133]; NA	ActiLife v.3.2 & MAHUffe v.1.9.0.3; SPSS v.26.0	Sleep, water activity
	51	Waistline on hip (Belt)	30, 15 sum to 60	Diary entries	7; 6h, 3d (NR)	Freedson (1998) [31]; NA	NR; SAS v.9.1	Bathe, sleep, swim
	67	R Hip (Elastic waist band)	30[†], 5	≥10 consec min of 0 CPM	7-14; 10h, 7d (NR)	Troiano (2008) [133]; NR	MAHUffe program; SPSS v.15.0	Bathe, contact sports, sleep, swim
	75	Hip around the waist (Belt)	30[†], 5[¥]	NR	7; NR, 3d (NR)	Freedson (1998) [31]; NA	NR; SPSS v.18	NR
GT1X (CD); CD	69	N-dom Hip (Belt)	CD, 60	NR	7; 8h, 3d (NR)	Troiano (2008) [133]; NA	NR; SAS PROC MI	NR
	70	N-dom Hip (Belt)	CD, 60	Troiano (2007) [160] algorithm	7; 8h, 1d (0d)	Troiano (2008) [133]; NA	SAS program; SAS v.9.4	NR

GT3X (2009); ACC (3)	4	Hip (Elastic belt)	NR$^{30-100}$, NR$^{1-60}$	NR	7; 8h, 3d (NR)	Freedson (1998) [31]; NA	NR; R v.3.4.3	Sleep, Water activity
	40	R Hip (Elastic belt)	NR$^{30-100}$, 60	Troiano (2007) [160] algorithm	7; 10h, 4d (NR)	Freedson (1998) [31]; NA	ActiLife v.6.12.0; SPSS v.20.0	Bathe, sleep, swim
	52	NR (NR)	NR$^{30-100}$, NR$^{1-60}$	Log entries	7; 6h, 3d (NR)	Freedson (1998) [31], Atkin (2012) [146]; NR	NR; SAS v.9.3	Bathe, sleep, swim
	60	Waist (Elastic belt)	NR$^{30-100}$, 60	NR	5-21; 0h, 5d (1d)	Troiano (2008) [133]; NR	ActiLife NR; NR	Contact sport, sleep, water activity
	62	R Hip (NR)	NR$^{30-100}$, NR$^{1-60}$	≥60 consec min of 0 CPM (≤2 min of 1-100)	7-14; 10h, 4d (0d)	Troiano (2008) [133]; Tudor-Locke (2008) [147]	ActiLife v.6.12.0; SPSS v.22.0	Bathe, sleep, swim
	63	R Hip (NR)	NR$^{30-100}$, 5 convert to 60	60 consec min of 0 CPM (≤2 min of 1-100)	7; 10h, 4d (0d)	Troiano (2008) [133]; NR	ActiLife v.6.12.0; SPSS v.22.0	Bathe, sleep, swim
	65	R Hip (Elastic belt)	100, 1 transform to 60	Cain (2017) [134] algorithm	7; each NR	Freedson (1998) [31]; NA	ActiLife v.6.6.2; SPSS v.20.0	Bathe, water activity
	72	Waist (NR)	NR$^{30-100}$, NR$^{1-60}$	NR	5; 6h, 3d (NR)	NR; NA	NR; SPSS v.NR	NR
	87	R Hip (Elastic belt)	NR$^{30-100}$, NR$^{1-60}$	Troiano (2007) [160] algorithm	7; 10h, 4d (NR)	Freedson (1998) [31]; NA	ActiLife v.6.11.3; SPSS v.21	Bathe, contact sports, sleep, swim
GT3X+ (2012)	34	R Hip at the iliac crest (Elastic belt)	NR$^{30-100}$, 15 sum to 60	Troiano (2008) [160] algorithm	7; 6h, 3d (NR)	Atkin (2012) [146], Kim (2015) [148]; NA	NR; SPSS v.24	Bathe, swim
	35	NR (NR)	NR$^{30-100}$, NR$^{1-60}$	NR	7; each NR	NR	NR; NR	NR
	41	NR (NR)	NR$^{30-100}$, NR$^{1-60}$	≥60 consec min of 0 CPM (1-2 min of 1-100)	7; NR, 4d (NR)	Troiano (2008) [133]; NR	ActiLife NR; SPSS v.18	NR
	58	R Hip (Elastic belt)	30, 60	≥60 consec min of 0 CPM (1 min "interruption")	7; 10h, 4d (NR)	Hagstromer (2010) [149], Hansen (2012) [150]; NR	NR; SPSS v.19	Bathe, sleep, swim
wGT3X-BT (2013); ACC (3)	2	N-dom Hip at iliac crest (NR)	100, 60	Choi (2011) [133] algorithm	7; 10h, 4d (1d)	Agiovlasitis (2022) [142]; NA	ActiLife v.6.13.4; SPSS v.28.0	Bathe, sleep (night-time)
	14	R Hip (Waist belt)	60, 60	NR	7; 10h, 1d (NR)	Troiano (2008) [133]; NA	NR; NR	Bathe, sleep, water activity
	15	Waist (Elastic belt)	NR$^{30-100}$, 60	≥60 consec mins of 0 CPM	5-6; 10h, 4d (NA)	Freedson (1998) [31]; NA	NR; SPSS v.24.0	Bathe, sleep (bedtime)
	21	N-dom Hip (Elastic belt)	60, 60	≥90 consec mins of 0 CPM (1-2 min of 0-100)	7; 8h, 3d (1d)	Troiano (2008) [133], Matthews (2008) [151]; NA	ActiLife v.6.13.3 & R package; R v.4.2.2	Bathe, contact sports, sleep, swim
	31	Waistline on R Hip at iliac crest (Waist strap)	60, 60	Troiano (2007) [160] algorithm	7; 10h, 4d (1d)	Atkins (2012) [146]; NA	ActiLife v.6.13.4; SPSS v.26	Bathe, sleep, swim
	33	Hip (Belt around the waist)	NR$^{30-100}$, 15 sum to 60	NR	7; 6h, 3d (NR)	Atkins (2012) [146]; NA	NR; SPSS v.21	Bathe, sleep, swim
	60	Waist (Elastic belt)	NR$^{30-100}$, 60	NR	5-21; 0h, 5d (1d)	Troiano (2008) [133]; NR	ActiLife v.NR; NR	Contact sport, sleep, water activity
	61	NR (NR)	30, 60	"60 min"	NR; 10h, 4d (NR)	Freedson (1998) [31]; NA	NR; R v.NR	NR

	89	Dominant Waist (Elastic belt)	100, 60	NR	7; 10h, 4d (NR)	Troiano (2008) [133]; NA	ActiLife v.6.13.1; SPSS v.25.0	Bathe, sleep, swim, water activity
GT9X (2014); ACC (3)	26	N-dom Wrist (Watch band)	30, 60	Troiano (2007) [160] algorithm	7; each NR	Freedson (1998) [31]; NA	ActiLife v.6.13.4; NR	Water activity (long duration)
NR (CD); CD	48	Waist (NR)	NR, NR	≥20 consec mins of 0 CPM	7-10; NR, 5d (2d)	Modified Freedson (1998) [31]; NA	From Wilson (2008); SAS v.9.0	Sleep
Apple Inc. (Los Altos, US)								
Series 4 (2019); HRM/ACC (3)	25	N-dom Wrist (Sports Band)	NR, NR	0 in hourly heart rate	7d; 10h (M1) or 1,703-24,369 steps/d (M2), 4d (0d)	NR; Tudor-Locke (2004) [152]	Excel; STATA v.17	Charge (nightly), sleep, swim (in open waters)
Axivity Ltd. (Newcastle upon Tyne, UK)								
AX3 (~2013); ACC (3)	18	Wrist (NR)	NR[12.5-3,200], NR	NR	7; NR, 7d (2d)	Esliger (2011) [32]; NA	GitHub (Link Provided); NR	Sleep
	38	R Thigh (Medical tape and Flexifix®)	NR[12.5-3,200], NR	NR	7; NR, 0d (NR)	NR; NR	ACTi4; R v.NR	NR
BodyMedia Inc. (Pittsburgh, US)								
SenseWear Armband (2007); ACC (2)	49	Upper R Arm (Elastic Velcro® strap)	32†, NR	NR	7; each NR	NR	Sensewear Pro v.6.2; SPSS v.18.0	Bathe, swim
CamNtech Ltd. and Inc. (Fenstanton, UK)								
ActiHeart (2003); ACC (3)/HRM	56	Chest (2 EKG pads at V_2 and V_5)	100†, 30	Activities ≤1.49 MET-min w/in sleeping HR zone	7; 5h, 7d (2d)	In MET-min: SB (≤1.49), LPA (1.5-2.99), MPA (3-5.99), VPA (≥6); NA	NR; SPSS v.20.0	Bathe or water activity
Fitbit International Limited (Dubin, IE) and LLC (Mountain View, US)								
Zip (2012); ACC (3)	43	Hip/Waist (Clip on belt loop/waist band, or in pocket)	100†, NR	NR	10-21; NR, NR (NR)	NR; NR	NR; NR	Before/after school activities
Flex (2013); ACC (3)/HRM	57	Wrist (Watch Band)	100†, NR	NR	7 or until stable; NR, 3d (NR)	NA; NR	NR; NR	Charge (every 2-3d for 30 min)
Flex 2 (2016); ACC (3)/HRM	85	Wrist (Watch Band)	100†, NR	NR	7; each NR	NA; Tudor-Locke (2009) [153]	NR; SPSS v.26	Sleep
Inspire 2 (2020); ACC (3)/HRM	53	Wrist (Watch Band)	100†, NR	NR	NR; each NR	NR	MATLAB R2023a; SPSS v.28.0.1.1	NR
Muscle Dynamics Fitness Networks (Torrance, US)								
Caltracs (CD); ACC (1)	81	Hip (Small pouch)	NR, NR	NR	7; NR, 5d (2d)	NR; NA	NR; SPSS v.9.0.1	Contact sport, bathe, sleep, swim
	82	Hip (Small pouch)	NR, NR	NR	3; each NR	NR; NA	NR; SPSS v.10.1.3	Bathe, sleep, swim
New Lifestyles Inc. (Lees Summit, US)								
NL-1000 (CD); PED/ACC (1)	36	Midway between umbilicus and side of body (Belt)	NR, 4¥	NR	14; NR, 4d (NR)	NA; Tudor-Locke (2004) [152]	NR; SPSS v.17	NR
	37	Midway between iliac crest and umbilicus (Belt)	NR, 4¥	NR	14; each NA	NA; NR	NR; SPSS v.15	Bath, sleep, swim, water activities
	59	NR (NR)	NR, 4¥	NR	NR; each NR	NA; 7,500 steps/d	NR; SPSS v.21	NR

Omron Healthcare Inc. (Kyoto, JP)								
HJ-700IT (2002); PED/ACC (1)	66	Waist (Clip on pants or pant pocket)	NR, NR	“No timestamp”	7; 10h, 5d (1d)	NA; Tudor-Locke (2004) [152]	NR; SPSS v. 14.0	Bathe, sleep (bedtime), swim
HJ 720ITC (2004); PED/ACC (1)	19	Waistline over hip (NR)	NR, 3,600	NR	7; 8-9h, 3d (NR)	NA; NR	OMRON HM & Excel; STATA v.11.2	Sleep
	20	NR (NR)	NR, NR	NR	7; 8h, 3d (NR)	NA; Tudor-Locke (2004) [152]	NR; STATA v.11	Sleep
PAL Technologies Ltd. (Glasgow, SC)								
activPAL (2001); ACC (3)/INC	24	Thigh (PALstickies™)	20†, 15¥	NR	7; NR, 5d (NR)	Chastin (2009) [154] & Tudor-Locke (2005) [155]; NR	NR; SPSS v.16	Bathe, swim
	88	R Mid-Thigh (Tegaderm®)	20†, 15¥	NR	7; NR, 4d (NR)	NR; NR	NR; SAS v.NR	NR
Polar Electro Oy (Kempele, FI)								
Ignite (CD); HRM	16	N-dom Wrist (Watch band)	NR (CD), 1	NR	9; 24h, 4d (NR)	NA; NA	FlowSync v.6.7.0; SPSS v.27.0	NA
Respironics Inc. (Murrysville, US)								
Actical Mini-Mitter (2008); ACC (1)	17	NR (NR)	32†, 30	Log	7; 10h, 4d (1d)	Heil (2006) [157]; NA	NR; SAS v.9.2	Sleep
ActiWatch-NR (CD); ACC (1)	61	NR (Watch band)	32†, 15¥	"60 min"	NR; 10h, 4d (NR)	Van Alphen (2020) [157]; NA	NR; R v.NR	NR
Stayhealthy Inc. (Monrovia, US)								
RX3 (~2003); ACC (3)	73	R Hip (Waist band)	$NR^{0.7\text{-}5000}$, 60	≥60 consec mins of ≤10 CPM (1-2 mins of 0-100)	8; 10h, 4d (NR)	Peiris (2016) [158]; NA	NR; SPSS v.22.0	Sleep, water activity
UCLA Wireless Community (Los Angeles, US)								
PAM System (CD); ACC (3)	30	R or L Ankle or Waist (NR)	40, 60	NR	14; each NR	NR; NA	PAM Server System; SPSS v.19.0	Bathe, water activity
Yamax Corp. of Yamasa Tokei Keiki Co., Ltd. (Tokyo, JP)								
Keep Walking LS2000 / Digiwalker SW-200 (2007); PED	5	Waist, align w/ R knee (Waist band)	NA	NR	7; 500 steps, 3d (NR)	NA; NR	NA; SPSS v.20	NR
	44	NR (NR)	NA	NR	7-NR; NR, 3d (NR)	NA; NR	NA; NR	NR
	80	Waist, align w/ knee (Waist band)	NA	NR	5; NR, 3d (NR)	NA; Tudor-Locke (2008/2011) [147,159]	NA; SPSS v.18	Sleep
Digiwalker SW-500 (CD); PED	76	Hip/Waist, align w/ mid-thigh (Belt/waist band)	NA	NR	7; NR, 7d (2d)	NA; NR	NA; NR	Sleep (bedtime)
	77	Hip/Waist, align w/ mid-thigh (Belt/waist band)	NA	NR	7; each NR	NA; NR	NA; NR	Bathe, sleep (bedtime), swim
	78	Hip/Waist, align w/ mid-thigh (Belt/waist band)	NA	NR	7; NR, 7d (2d)	NA; NR	NA; NR	Bathe, sleep (bedtime), swim
	79	Hip/Waist, align w/ mid-thigh (Belt/waist band)	NA	NR	7; each NR	NA; Tudor-Locke (2004) [152]	NA; NR	Bathe, sleep (bedtime), swim
Keep Walking LS7000 / Digiwalker, SW-700 (CD); PED	10	R Waist (NR)	NA	NR	4; NR, NR (0)	NA; Tudor-Locke (2004) [152]	NA; NR	Bathe, swim
	71	NR (NR)	NA	NR	2; NR, NR (0)	NA; NR	NA; SPSS v.25.0	NR

	77	Hip/Waist, align w/ mid-thigh (Belt/waist band)	NA	NR	7; each NR	NA; NR	NA; NR	Bathe, sleep (bedtime), swim
	78	Hip/Waist, align w/ mid-thigh (Belt/waist band)	NA	NR	7; NR, 7d (2d)	NA; NR	NA; NR	Bathe, sleep (bedtime), swim
	79	Hip/Waist, align w/ mid-thigh (Belt/waist band)	NA	NR	7; each NR	NA; Tudor-Locke (2004) [152]	NA; NR	Bathe, sleep (bedtime), swim
	83	NR (NR)	NA	NR	7; each NR	NA; Tudor-Locke (2004) [152]	NA; SPSS v.12.0	Bathe, sleep, swim
	84	NR (NR)	NA	NR	7; each NR	NA; Tudor-Locke (2004) [152]	NA; NR	NR
	90	NR (NR)	NA	NR	5; each NR	NA; NR	NA; SPSS v. 21.0	Before/after program
NR (CD); PED	9	NR (NR)	NA	NR	7; each NR	NA; NR	NA; NR	NR
	47	NR (NR)	NA	NR	NR; each NR	NA; NR	NA; NR	NR

Notes. f_s: sampling frequency; ID: study identification number (see Table A2).

† Not explicitly reported in the included study. However, according to the manufacturer, only one sampling frequency can be set.

¥ Not explicitly reported in the included study. However, according to the manufacturer, only one epoch length can be set.

Table S2. Subjective techniques used to quantify physical activity and sedentary behavior in the included studies.

Tool Version	Elicited PA/SB Dimensions	ID	Items	Recall Days	Adaptions to the Tool	Reporter(s)
Interview						
PACI-MVPA	Frequency, Intensity	3	144^{I}	1^{R}	Included 24 activities; walk, jog, or run only if for a sustained exercise/play time	PPT
Author-Derived	Duration, Frequency,	23	CD	28^{R}	NR	PPT
	Intensity, Mode	91	CD	7^{R}	NR	PPT, CG
	Mode, Intensity	24	CD	7^{R}	NR	PPT
Log / Diary						
Author-Derived	Duration, Volume (Steps#)	36	42^{I}	14^{A}	NR	CG
		37	42^{I}	14^{A}	NR	CG
	Frequency	58	28^{I}	7^{A}	NR	CD
	Mode, Other (e.g., wear	1	672^{I}	3^{A}	NR	RS, CG
	times)	25	223	7^{A}	NR	PPT, CG, RS
		26	CD	7^{A}	NR	PPT, CG
	Mode, Volume (Steps#)	76	CD	7^{A}	NR	CG
	Volume (Steps#)	5	7^{I}	7^{A}	NR	NR
		9	7^{I}	7^{A}	NR	PPT, CG*
		10	4^{I}	4^{A}	NR	RS
		44	7-NR^{I}	7-NR^{A}	NR	PPT
		47	7^{I}	7^{A}	NR	PPT, CG
		59	7^{I}	NR ^{A}	NR	CD
		71	2^{I}	2^{A}	NR	CD
		77	7^{I}	7^{A}	NR	PPT
		78	7^{I}	7^{A}	NR	PPT, CG
		79	7^{I}	7^{A}	NR	PPT, CG
		80	7^{I}	7^{A}	NR	PPT, CG
		83	7^{I}	7^{A}	NR	PPT, CG
		84	7^{I}	7^{A}	NR	PPT
		90	5^{I}	5^{A}	NR	CD
	Other (e.g., wear times)	17	CD	7^{A}	NR	CG
		46	CD	7^{A}	NR	PPT, CG
		51	CD	7^{A}	NR	PPT
		52	CD	7^{A}	NR	PPT, CG
		67	CD	7-14^{A}	NR	PPT, CG
		88	CD	7^{A}	NR	CG
		89	CD	7^{A}	NR	CG
Bouchard's PAR	Intensity, Mode	16	CD	8^{A}	Y/N items; added examples, room to provide more info	PPT, CG
		81	CD	7^{A}	Used every minute each day	RS
		82	CD	3^{A}	Used 10 times each day	CG
Based on Mâsse (1999) [145]	Duration, Frequency, Intensity, Mode, Other	27	CD	7^{A}	PPT: TV shows, exercise, or walking bouts items only	PPT, CG, RS
Questionnaire						
Author-Derived	Duration	39	1^{I}	1^{G}	NR	CG, PPT
FFAS	Duration, Frequency	54	CD	7^{G}	Deleted unclear and low-response-rate items	PPT
		55	CD	7^{G}	Deleted unclear and low-response-rate items	PPT
GLTEQ	Frequency	11	3	7^{R}	NR	CG
		12	3	7^{R}	NR	CG
		13	3	7^{R}	NR	CG
IPAQ-SF	Duration, Frequency	18	7	7^{R}	Added pictures of activities and time	PPT, CG
		28	7	7^{R}	NR	PPT
		33	7	7^{R}	NR	PPT, CG
		35	7	7^{R}	NR	PPT
		45	7	7^{R}	Changed to proxy's POV of PPT	CG
		46	7	7^{R}	NR	PPT, CG
		51	7	7^{R}	NR	PPT, CG

		52	7	7[R]	NR	PPT, CG
		56	7	7[R]	NR	CG
		65	7	7[R]	NR	PPT
		68	7	7[R]	NR	PPT, CG
		75	7	7[R]	NR	PPT
IPAQ-PR	Duration	72	189	7[A]	NR	CG
IPAQ-NR	Duration, Frequency	8	7-12	7[R]	NR	PPT
		44	7-12	7[R]	NR	PPT
		49	7-12	7[R]	NR	PPT
		64	7-12	7[R]	NR	PPT
NHANES-III	Duration, Frequency	22	CD	28-31[R]	NR	PPT, CG
		29	CD	28-31[R]	Focused on walking activities	PPT, CG
		78	CD	28-31[R]	Added SO involvement	PPT, CG
SLÁN 2009	Duration, Frequency	87	NR	NR	Added pictures for activities and intensities	PPT
SBQ-ID	Duration	86	24	7[G]	Rephrased questions, swapped to open answers, added examples, split weekend into each day	PPT
NR	CD	32	CD	NR	NR	PPT
		53	CD	NR	NR	PPT
	Duration	74	CD	2[CD]	NR	CG

Notes. CD: cannot determine; CG: caregiver; ID: study identification number (see Table A2); FFAS: French Federation for Adapted Sport Survey; GLTEQ: Godin Leisure-time Exercise Questionnaire; IPAQ-SF: International Physical Activity Questionnaire–Short-Form; International Physical Activity Questionnaire–Proxy-Report; International Physical Activity Questionnaire–Unspecified Version; NHANES-III: National Health and Nutrition Examination Survey–Third Edition; NR: not reported; PACI-MVPA: Physical Activity Checklist Interview for Moderate-to-Vigorous Intensity; PAR: Physical Activity Record; POV: point of view; PPT: participant; SLÁN 2009: Survey of Lifestyle, Attitudes, and Nutrition in Ireland in 2009; SBQ-ID: Sedentary Behavior Questionnaire–for Adults with Intellectual Disability; RE: researcher.

* as needed

[A]: Assessment timeframe

[R]: Recall timeframe

[G]: General timeframe (e.g., “a week,” “regularly”)

[I]: Inferred
